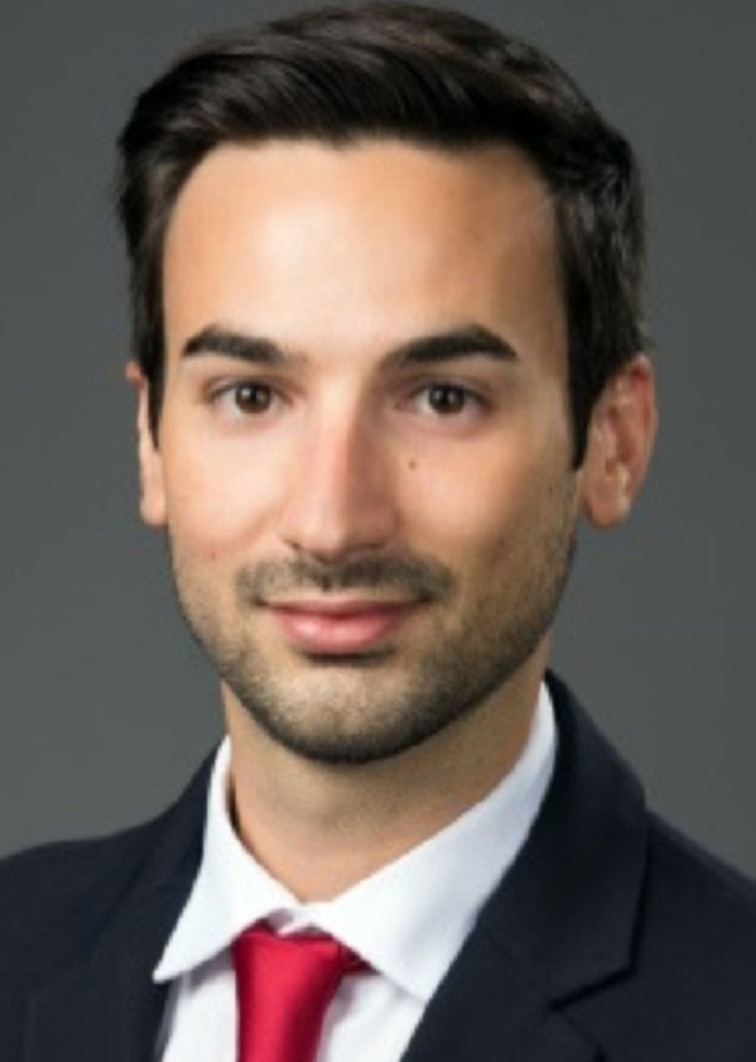# Mutual Prodrugs of 5‐Fluorouracil: From a Classic Chemotherapeutic Agent to Novel Potential Anticancer Drugs

**DOI:** 10.1002/cmdc.202100473

**Published:** 2021-09-07

**Authors:** Valeria Ciaffaglione, Maria N. Modica, Valeria Pittalà, Giuseppe Romeo, Loredana Salerno, Sebastiano Intagliata

**Affiliations:** ^1^ Department of Drug and Health Sciences University of Catania Viale A. Doria 6 95125 Catania Italy

**Keywords:** Mutual prodrugs, 5-Fluorouracil, 5-FU hybrids, 5-FU conjugates, Anticancer agents

## Abstract

The development of potent antitumor agents with a low toxicological profile against healthy cells is still one of the greatest challenges facing medicinal chemistry. In this context, the “mutual prodrug” approach has emerged as a potential tool to overcome undesirable physicochemical features and mitigate the side effects of approved drugs. Among broad‐spectrum chemotherapeutics available for clinical use today, 5‐fluorouracil (5‐FU) is one of the most representative, also included in the World Health Organization model list of essential medicines. Unfortunately, severe side effects and drug resistance phenomena are still the primary limits and drawbacks in its clinical use. This review describes the progress made over the last ten years in developing 5‐FU‐based mutual prodrugs to improve the therapeutic profile and achieve targeted delivery to cancer tissues.

## Graphical abstract

**New and improved**: 5‐Fluorouracil (5‐FU) is one of the most commonly used chemotherapeutic agents; however, its use is limited by unfavorable physicochemical properties and high nonspecific cytotoxic effects. Therefore, to overcome specific drawbacks of 5‐FU and to enhance its therapeutic efficacy, several 5‐FU‐based mutual prodrugs have been developed in recent years for both improving biological activity and achieving targeted delivery to cancer tissues.

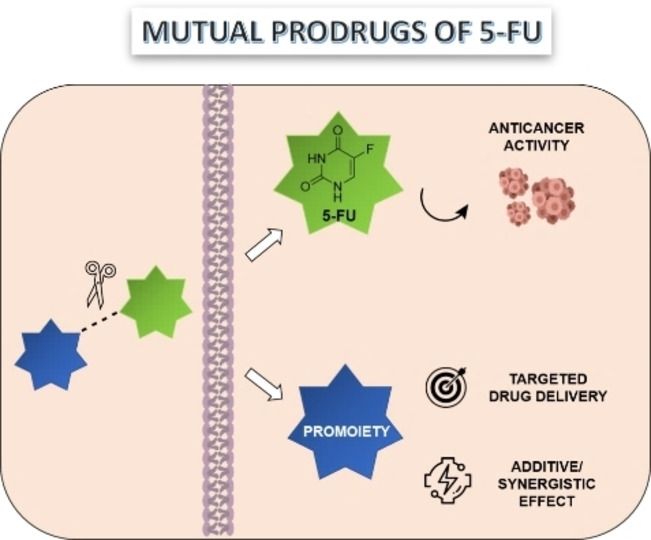

## 1 Introduction

**Figure 1:**
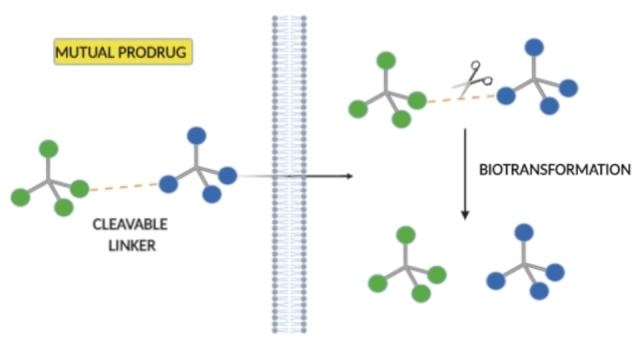
A simplified illustration of the mutual prodrugs approach.

Prodrug strategies have attracted much interest from the scientific community in the challenging drug development program. One of the most significant issues to face in introducing new drugs in the market is the low pharmacokinetic and pharmacodynamic profile of candidate molecules. Since the introduction of the prodrug concept by Adrian Albert in 1958,^[1]^ this medicinal chemistry strategy has become a top‐rated tool in the drug discovery process. Nearly 10 % of all commercialized drugs belong to this category, with at least 30 prodrugs approved by the Food and Drug Administration (FDA) in the last decade.^[2]^ The term “prodrug” refers to molecules with little or no biological activity as such that become pharmacologically relevant upon activation. The administered prodrug can then be enzymatically or chemically processed before exerting its pharmacological activity.^[3]^ This strategy allows overcoming undesirable physicochemical properties, including low water solubility, stability, affinity for cell transporters, which are necessary for absorption, distribution, metabolism, and excretion (ADME). This versatile approach can be achieved through different types of chemical entities. Indeed, prodrugs can be divided into two categories: (1) carrier‐linked prodrugs, consisting of an active compound bound to a linker, and (2) bio‐precursors, which involve compounds that undergo structural changes and are metabolized into new active chemical entities.^[4]^ Depending on the carrier introduced into the drug's structure, the first‐mentioned sub‐class can be further divided as follow: double, macromolecular, site‐specific, or mutual prodrugs. In general, mutual prodrugs, also known as “codrugs”, are made by two or more pharmacophores covalently linked, directly or through a cleavable spacer (Figure 1).^[5]^ The main advantage of mutual prodrugs is that upon administration and bioconversion, the two (or more) active molecules released can act at the same level (the target tissue), exploiting a synergistic effect. In some cases, the second pharmacophore exploits a different biological effect, providing additional benefits to the parent compound, or can be used as carrier to target the parent drug to a specific site of action. On the other hand, this strategy also allows to overcome issues related to the absorption of two different drugs administrated separately by conjugating them into the same chemical entity with improved pharmacokinetics.

The first example of a mutual prodrug with the clinical application was sulfasalazine, which was approved for the treatment of ulcerative colitis.^[6]^ This drug is constituted by sulfapyridine linked to the anti‐inflammatory 5‐amino salicylic acid through the azo linkage (Table 1). Sulfasalazine represents an example of a directly‐coupled mutual prodrug, which is metabolized by intestinal bacteria that allow the release of the two active molecules after cleavage exerted by the action of azoreductase enzymes.^[7]^

**Table 1:** Examples of mutual prodrugs and their purpose.

Mutual prodrugs	Linker	Activation	Purpose	Ref.
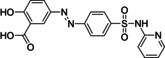				
sulfasalazine	Azo linkage	Azoreduction	Site‐specific drug delivery (colon‐specific action)	[7]
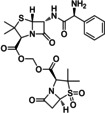				
sultamicillin	Ester linkage	Hydrolytic cleavage	To improve oral absorption Synergistic activity (antibiotic)	[10]
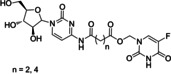				
5‐FU/Cytarabine mutual prodrug	Acyloxy‐ methylene linkage	Hydrolytic cleavage	Synergistic activity (anticancer)	[11]
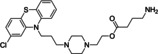				
GABA/Perphenazine mutual prodrug	Ester linkage	Hydrolytic cleavage	To minimize side effects (extrapyramidal)	[12]
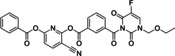				
emitefur	Ester linkage	Hydrolytic cleavage	To potentiate the antineoplastic activity	[13]

Similarly, the mutual prodrug approach has been applied to several classes of compounds by taking advantage of different functional groups or linkers susceptible to hydrolysis (Table 1). However, the concept of the mutual prodrug is different from that of “hybrid” or “conjugated” drug, where the two pharmacophores are bound permanently, and no cleavage occurs after administration.^[8]^ Mutual prodrugs might found applications for several purposes, such as reducing collateral effects, improving pharmacokinetic, giving an additional pharmacological activity to the parent drugs, or achieving synergistic action.^[9]^ Although the mutual prodrug approach gave advantages for drug optimization, several limitations still need to be faced. Firstly, this strategy requires specific functional groups for linkage, and a careful choice of the carrier is also needed. Ideally, the carrier should be nontoxic, non‐immunogenic, non‐antigenic, chemically stable, and should not be accumulated into the body after hydrolysis.

**Figure 2:**
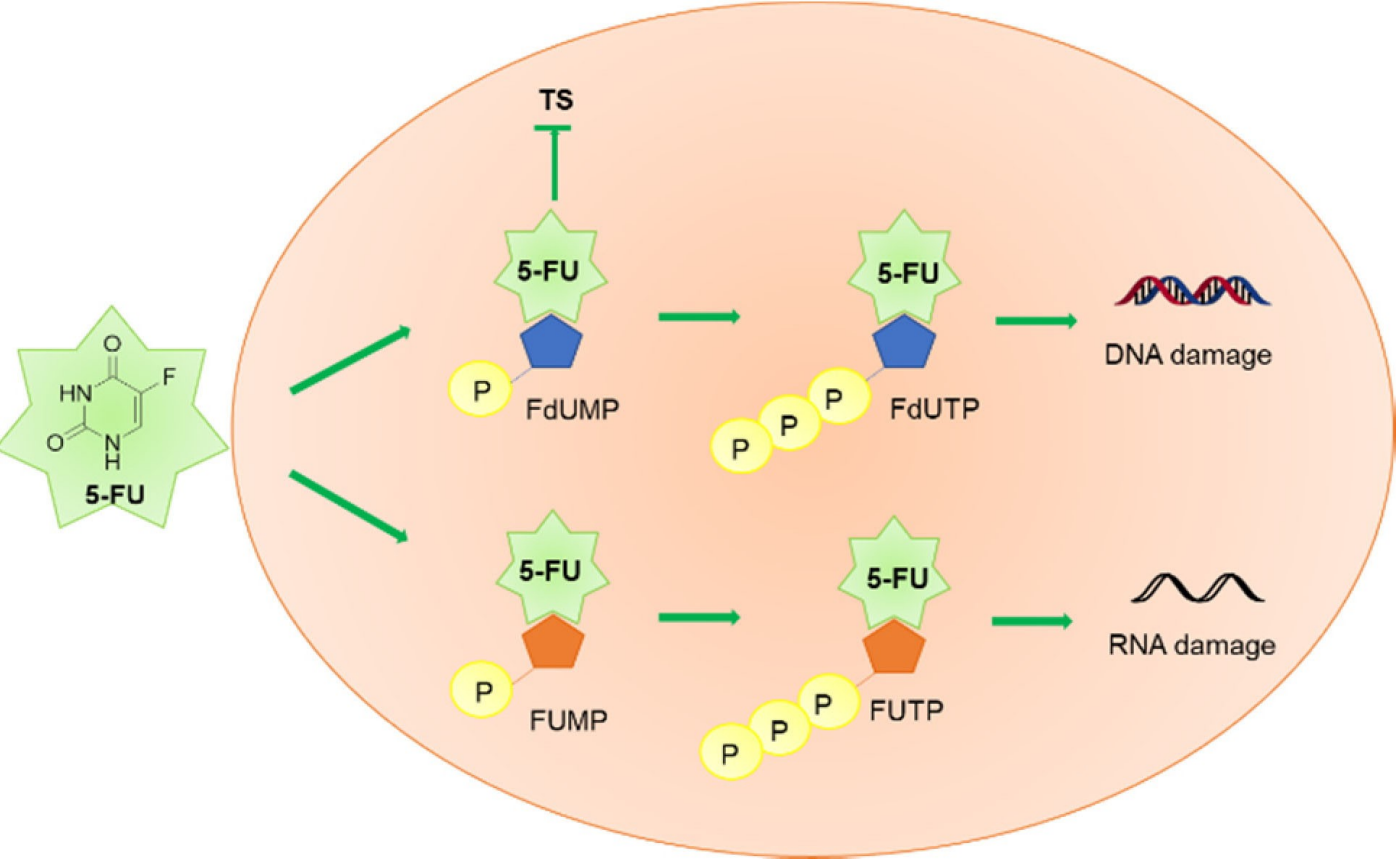
Schematic representation of 5‐FU metabolism leading to active metabolites FdUMP, FdUTP, and FUTP which cause DNA and RNA damage.

Amongst the wide variety of codrugs developed so far, a growing interest has been focused on 5‐fluorouracil (5‐FU) mutual codrugs, which are the object of this review. Indeed, 5‐FU represents a backbone in chemotherapy and finds in this strategy a tool to improve its clinical usage. Specifically, 5‐FU is an antineoplastic agent belonging to the class of antimetabolites. It is a nucleobase analog of uracil, bearing a fluorine atom at position 5 of the pyrimidine heterocycle, widely used for the treatment of several solid tumors, including those involving the gastrointestinal (colon, pancreas, stomach) and genitourinary (ovary, prostate) systems.^[14]^ The mechanism of cellular uptake and the mode of action of 5‐FU have been elucidated.^[15]^ It has been proven that 5‐FU is rapidly taken up by the cells, taking advantage of the uracil transporter.^[16]^ To exert its cytotoxic properties, 5‐FU must be converted into three active metabolites: fluorodeoxyuridine monophosphate (FdUMP), fluorodeoxyuridine triphosphate (FdUTP), and fluorouridine triphosphate (FUTP) (Figure 2). These metabolites are responsible for the antineoplastic activity of 5‐FU because they inhibit the nucleotide synthetic enzyme thymidylate synthase (TS) and RNA synthesis. In particular, FdUTP and FUTP are incorporated into the DNA and RNA structures, respectively, altering the DNA synthesis, and blocking the mRNA translation.^[17]^

Furthermore, flow cytometric studies showed that the cellular injury caused by 5‐FU influences the cell growth modulation, with three main effects, including loss or accumulation of S phase cells, G2/M block, and G1‐S arrest.^[18]^ Nonetheless, most of the administered 5‐FU is converted into dihydrofluorouracil (DHFU) and inactivated in the liver by the rate‐limiting enzyme of this process, dihydropyrimidine dehydrogenase (DPD). This enzyme plays a crucial role in the development of 5‐FU resistance.^[19]^ Indeed, the relationship between responsiveness to 5‐FU, and the mRNA expression of the enzymes involved in 5‐FU metabolism was investigated. In particular, high levels of DPD and thymidine phosphorylase (TP) mRNAs showed to be related to metastatic liver tumors and reduced sensitivity to 5‐FU. Also, microRNAs (miRNAs) might play a potential role in promoting 5‐FU resistance to colorectal cancer.^[20]^ Although 5‐FU is used as first‐line treatment for several solid tumors and adjuvant chemotherapy,^[21]^ its clinical use is limited by several drawbacks, with the drug resistance, short half‐life (10–20 minutes), and nonspecific cytotoxic actions remaining the most common ones.^[22]^ Moreover, the most frequent side effects of 5‐FU include myelosuppression, cardiotoxicity, gastrointestinal, neurological, and dermatological disorders.^[23]^ With this in mind, several 5‐FU derivatives have been developed to counteract specific limitations in the clinical use of 5‐FU; mainly, aiming to reduce the off‐target toxicity, the drug resistance, or improve the cytotoxicity in the site of action.^[24]^ In this field, it is worthwhile to mention a 5‐FU mutual prodrug named emitefur (Table 1), derived from the combination of 1‐ethoxymethyl‐5‐FU and 3‐cyano‐2,6‐dihydroxypyridine (CNDP), a DPD inhibitor.^[25]^ This conjugate demonstrated to easily release 5‐FU and CNDP,^[13]^ and was a valid mutual prodrug candidate submitted to clinical trials for colorectal cancer.^[26]^ Although the final unsuccessful results due to severe side effects, emitefur represented an attempt of developing a 5‐FU‐based mutual prodrug with increased anticancer activity, thanks to the inhibition of 5‐FU degradation.^[27]^ Herein, we summarized the significant advances in the development of 5‐FU mutual prodrugs over the past decade.

## 2 Mutual Prodrugs of 5‐FU with Improved Biological Activity

Growing evidence has shown improved anticancer activity by coadministration of 5‐FU with various other chemotherapeutic agents, from naturally occurring molecules to approved anticancer drugs.^[28]^ On this basis, the design of 5‐FU‐based mutual prodrugs that combine two active pharmacophoric moieties in a single chemical entity is of particular interest in medicinal chemistry. The conjugation of 5‐FU with other well‐known anticancer drugs has been performed with the primary purpose of improving the pharmacological profile of the 5‐FU, gaining a synergistic/additive effect. Notably, the introduction of cleavable linkers, such as carbamate, succinyl, glutaryl, or diamines, is essential for releasing the two biologically active moieties after administration. The current section describes studies on the development of 5‐FU‐based mutual prodrugs showing several beneficial effects, such as increased cytotoxicity towards malignant cells, reduced systemic side effects or drug resistance.

### 2.1 5‐FU/HDAC inhibitors

**Figure 3:**
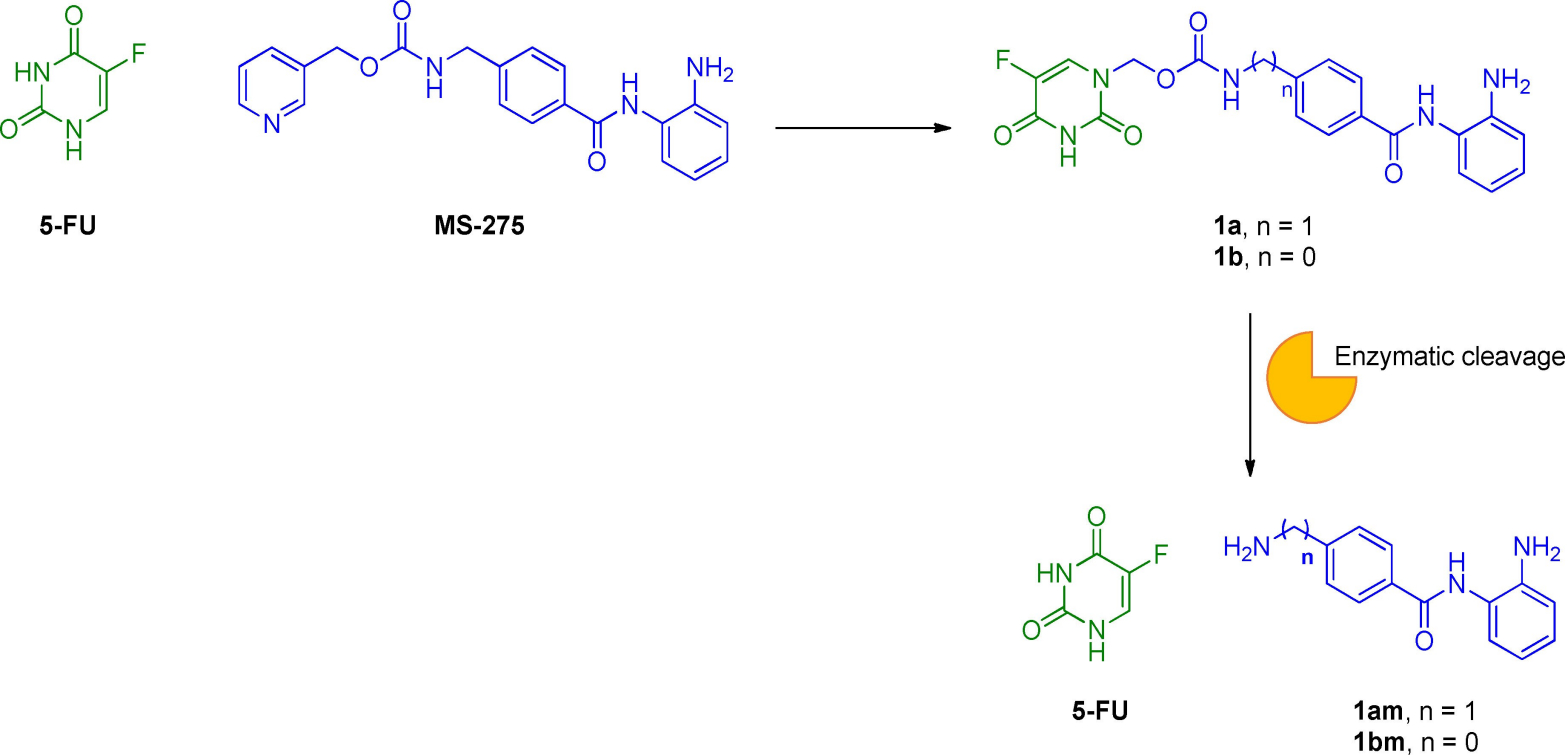
Chemical structures of 5‐FU, MS‐275, **1 a**,**b** and hydrolysis of the carbamate linker.

The inhibition of histone deacetylases (HDACs) has emerged as a novel therapeutic strategy in cancer research.^[29]^ HDACs are a family of 18 enzymes that catalyse the acetyl group‘s deletion from lysine residues within histones.^[30]^ Four classes of human HDACs have been identified, including Zn^2+^‐dependent (classes I, II, and IV HDACs) and NAD^+^‐dependent enzymes (class III HDACs). The histone acetylation/deacetylation dynamic process plays a pivotal role in epigenetic modulation of gene expression and chromatin remodeling.^[31]^ For this reason, deregulation of this tightly controlled process may be responsible for the generation of different kinds of tumors.^[32]^ The potential impact of HDACs on the aberrant acetylation state of histone proteins led to the development of HDAC inhibitors (HDACIs) as promising chemotherapeutic agents.^[33]^ Although the FDA has approved only four HDAC inhibitors to date, many derivatives have been synthesized.^[34]^ Structurally, they have been divided into four classes: *i)* hydroxamic acids; *ii)* short chain fatty acids, *iii)* benzamides; *iv)* cyclic tetrapeptides.^[35]^ Interestingly, many HDAC inhibitors showed relevant antineoplastic effects, especially when administered in combination with other anticancer therapies, although their mechanism of action depends on the tumor tissue and dosage.^[36]^ In general, their activity against cancer cells is related to the ability to block the cell cycle. Entinostat (MS‐275) belongs to the benzamide HDACIs class. It is a potent orally available drug, classified as a selective inhibitor of HDACs I and III.^[37]^ Many studies showed that MS‐275 exerts proapoptotic effects and blocks the cell cycle in G1‐phase by upregulating p21 and p53 proteins.^[38]^ Also, a significant induction of reactive oxygen species (ROS) was reported at high concentrations (e. g., 5 μM), followed by mitochondrial injury and caspase activation.^[39]^ Preclinical and clinical trials involving a combined use of MS‐275 with other anticancer drugs have been performed.^[40]^ In this context, a synergistic interaction between MS‐275 and the cytotoxic agent 5‐FU was proved in colorectal carcinoma (CRC) cell lines SW48, HT‐29, and Colo‐205.^[41]^ Interestingly, a combination of 5‐FU with MS‐275 led to a reduction of IC_50_ for 5‐FU from 2.6 to 6.5‐folds *in vitro*. This synergistic interaction might be explained by the increased apoptotic process due to the mutual enhancement of cytotoxicity in comparison with the drugs administered alone. For this reason, the conjugation of MS‐275 and 5‐FU structures in a new single chemical entity has been regarded as a promising approach in clinical use for reducing the dosage and the consequent side effects. To develop new multitarget anticancer drugs, Jiang and co‐workers combined the MS‐275 pharmacophore with 5‐FU, leading to new carbamate‐based codrugs **1 a,b** (Figure 3).^[42]^

The coupling reaction between the two bioactive entities was achieved by substituting the pyridine cycle of MS‐275 with 5‐FU, taking advantage of both 5‐FU antineoplastic effects and increased water‐solubility. Similar to other carbamate‐based prodrugs,^[43]^ the carbamate spacer was selected as a cleavable linker able to release both the HDACIs **1 am**,**bm** and the 5‐FU promoiety. Surprisingly, compounds **1 am** resulted very stable in artificial gastric juice, artificial intestinal juice, and human plasma during the *in vitro* stability assessment. On the other hand, the HDAC inhibitory activity of codrugs **1 a**,**b** (IC_50_=5.92 and 2.31 μM, respectively) was comparable with that of MS‐275 alone (IC_50_=2.09 μM). Nevertheless, **1 a**,**b** displayed less potent cytotoxic activity in K562, A549, U266, PC‐3, HCT‐116, ES‐2, and HL‐7702 cell lines activities than MS‐275, most likely due to the inability to release the two active promoieties. In addition, the influence of different routes of compound administration on the efficacy was not considered due to the lack of *in vivo* experiments. Future studies need to be performed to assess the hydrolysis of the carbamate linker *in vivo* and to evaluate the cytotoxic activity of the released 5‐FU, and **1 am**,**bm** moieties.

### 2.2 5‐FU/deoxypodophyllotoxin

Deoxypodophyllotoxin (DPT) is a naturally occurring compound belonging to the family of flavonolignans, which gained attention in traditional herbal medicine for its beneficial effects.^[44]^ It is isolated from *Podophyllum* plants along with other lignan derivatives, including its parent compound podophyllotoxin (PPT).^[45]^ DPT has been recognized as a potential therapeutic agent for its antitumor, anti‐inflammatory, antiviral, and antiallergic properties, although it has not found clinical application yet.^[46]^ In order to overcome its undesirable systemic toxicity and to improve its biological activity, structural changes were attempted, and several DPT analogs were developed. Among them, we can mention etoposide, its water‐soluble prodrug etoposide phosphate, and teniposide, clinically used for both solid and blood tumors.^[47]^ The mechanism of action of DPT has been studied, revealing that the inhibition of cell growth is due to the DPT's ability to suppress tubulin polymerization and the G2/M phase of the cell cycle.^[48]^ The pro‐apoptotic activity of DPT is related to the interference with several pathways and signaling molecules, such as p53 tumor suppressor, the apoptotic regulator Bax, and the protease enzymes caspases‐3 and ‐7.^[49]^

To improve DPT's pharmacological properties, Huang and co‐workers developed DPT and 5‐FU conjugates as potential mutual prodrugs.^[50]^ Their idea was inspired by previously reported hybrids of PPT with other anticancer drugs, such as camptothecin and vinorelbine.^[51]^ The first series of DPT‐5‐FU conjugates **4 a**–**f**, **5** is shown in Table 2.^[50a]^ These conjugates were obtained by coupling 4’‐demethy‐4‐deoxypodophyllotoxin (DDPT), which exerts a biological activity similar to that of DPT *in vivo*, with a functionalized 5‐FU moiety, such as 5‐fluorouracil‐*N* ^1^‐yl acetic acid (**2**) or *N*‐(5‐fluorouracil‐*N* ^1^‐yl acetic)‐amino acids (**3 a**–**f**), to gain advantage from the synergistic or additive pharmacological effects of both DDPT and 5‐FU.

**Table 2:** Antiproliferative activities and chemical structure of DDPT/5‐FU conjugates **4 a**–**f**, **5** and their precursors DDPT, **2** and **3 a**–**f**.

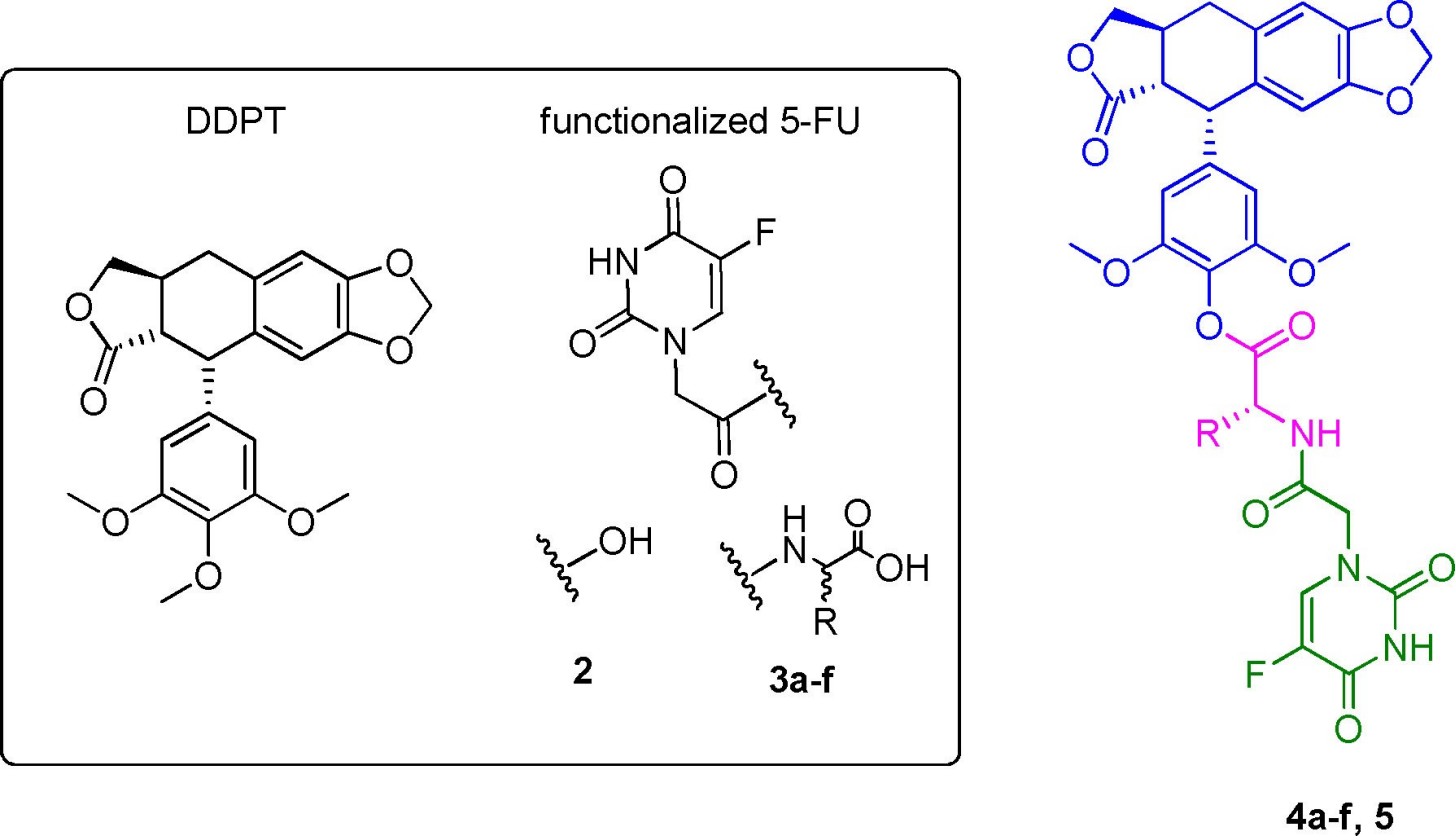						
Compd.	Config.	R	Cytotoxicity [IC_50_, μM]^[a]^			
			HL‐60	A‐549	HeLa	SiHa
**4 a**	*L*‐	Me	0.19	1.4	0.2	0.6
**4 b**	*L*‐	CHMe_2_	0.14	0.36	1.23	1.65
**4 c**	*L*‐	CH_2_CHMe_2_	0.063	1.07	0.43	0.35
**4 d**	*L*‐	CH_2_Ph	0.023	0.56	0.83	0.76
**4 e**	*L*‐	CH_2_CH_2_SMe	0.23	0.83	0.78	0.36
**4 f**	*D*‐	CH_2_CH_2_SMe	0.94	2.6	1.97	1.51
**5**	–	–	0.035	0.66	0.18	0.11
**DDPT**	–	–	2.96	1.8	53.3	43
**5‐FU**	–	–	68.3	54.8	82.2	218

[a] Data from Ref. [50a].

Amino acid linkers have been introduced to allow the coupling between the two parent drugs, DDPT and 5‐FU, and were chosen for their water solubility and nontoxic nature. *In vitro* assays were achieved to investigate their biological activity on four human tumor cell lines (i. e., promyelocytic leukemia HL‐60, lung carcinoma A‐549, cervical carcinoma HeLa, and cervical squamous cell carcinoma SiHa), while non‐tumor cells lines were not selected. In general, most of the new potential codrugs showed higher toxic activity than their parent compounds DDPT and 5‐FU, with **4 d** being the most promising. Structure‐activity relationship studies (SARs) revealed that the antiproliferative activity was strongly dependent on both the substituent at the α‐carbon of the amino acid and the amino acids′ configuration (e. g., *L*‐ was preferred). The ester functional group of **5** also improved the cytotoxicity compared to DDPT.^[52]^ The most interesting compound of this series, **4 d**, was further studied in A549 cells to better understand its effects on the cell cycle. As a result, **4 d** showed a pro‐apoptotic role due to its ability to block the cell cycle in the G2/M phase and activate caspase‐3 and ‐7, which are downstream effectors of apoptosis. Unfortunately, despite these promising preliminary results on the cytotoxicity of **4 d**, the selectivity towards cancer cells was not explored.

**Figure 4:**
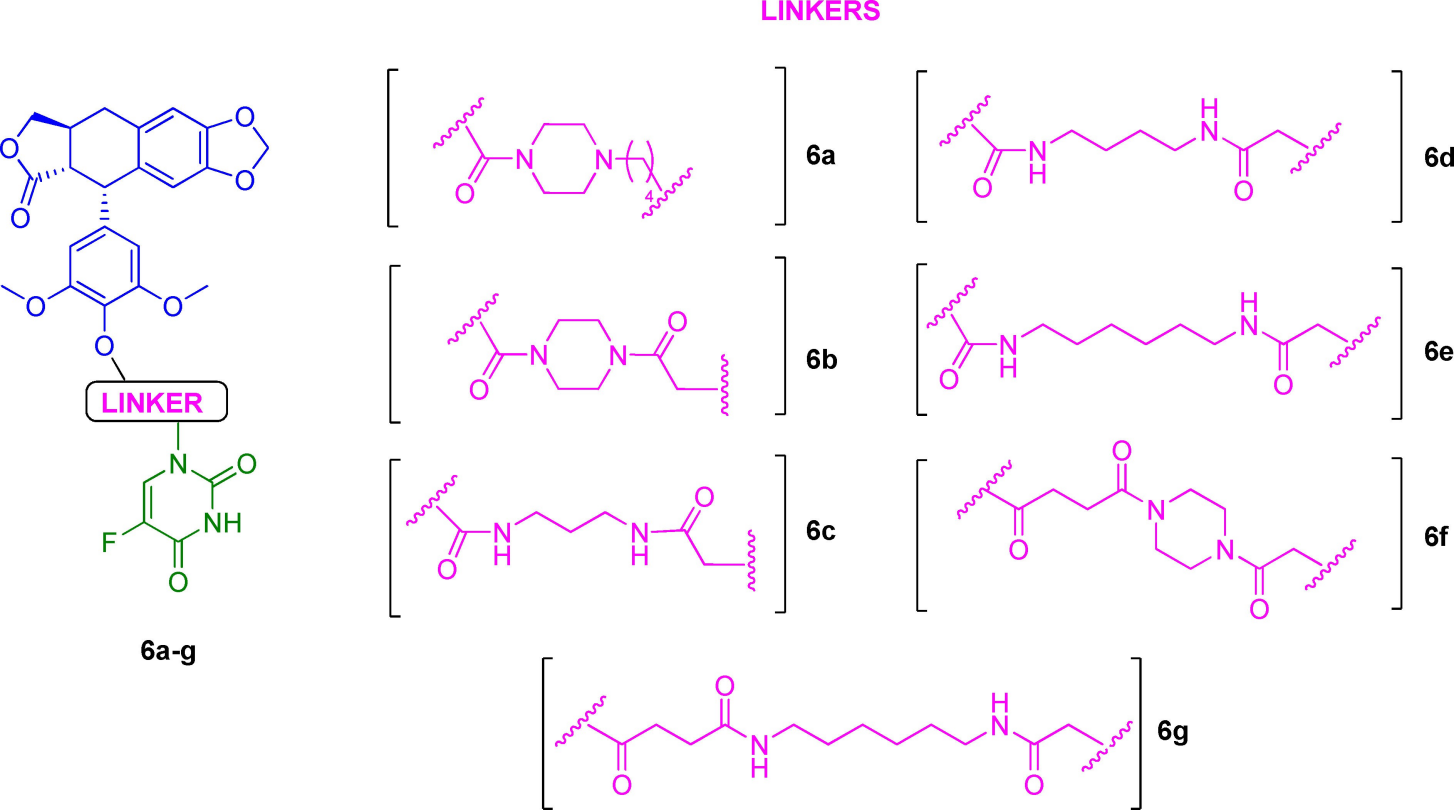
Chemical structures of DDPT‐5‐FU conjugates **6 a**–**g**.

Another series of DDPT‐5‐FU conjugates **6 a**–**g** (Figure 4) was developed in 2016 to extend the previous one with further structural changes and reduced side effects in normal cells.^[50b]^ For this purpose, diamines were used as linkers since these spacers have been demonstrated to be useful for balancing the hydrophilic/hydrophobic drug state *in vivo*.^[53]^ Conjugates **6 a**–**g** were studied in cancer (HepG2, A549, HeLa, and HCT‐8) and normal cells (WI‐38). It was found that their cytotoxic activity depended on the cell line, with the best results obtained in A549 cells. A general improvement of cytotoxicity in comparison to the parent pharmacophoric units DDPT and 5‐FU was confirmed. In addition, reduced toxicity in non‐tumorigenic cells was assessed.

Another interesting result was that the linker strongly influenced the biological activity of the codrug candidates. Indeed, higher toxicity was obtained when succinic acid was used as a spacer, in comparison with those analogs achieved through carbamate condensation (**6 f** *vs*. **6 b**, A549 IC_50_=1.35 and 2.41 μM, respectively). These findings suggest that future efforts should be focused on the development of new codrugs through this suitable linker. The proapoptotic activity of the most potent compound of the series (**6 g**) was further studied, showing to involve several signaling molecules, mainly cyclin B1, cdc2, and p‐cdc2 in A549 cells. Finally, compound **6 g**, which was found even more cytotoxic than etoposide, revealed potent anti‐angiogenic and vascular disrupting actions in human umbilical vein endothelial cells (HUVECs).^[54]^ On the whole, these results supported the synergism between DPT and 5‐FU; however, further investigations should be carried out to determine the full potential of this approach.

### 2.3 5‐FU/ubenimex

Ubenimex is a competitive protease inhibitor with immunomodulatory and antineoplastic properties, as shown in different types of tumors, such as leukemia, bladder, prostate, gastric and non‐small cell lung cancer.^[55]^ Ubenimex's contribution against tumorigenesis is mainly due to its ability to inhibit aminopeptidase N (CD13/APN), which has been regarded as a potential therapeutic target for anticancer treatments.^[56]^ Indeed, CD13 is a zinc‐dependent ectoenzyme with a leading role in tumor growth, angiogenesis, and metastasis, which is overexpressed in inflammatory disorders and tumors.^[57]^ In recent years, growing pieces of evidence have highlighted the potential benefits of introducing CD13 inhibitors in adjuvant therapy protocols. For example, in 2016, Yamashita and co‐workers studied the synergistic anticancer activity of ubenimex with other traditional antineoplastic drugs, such as 5‐FU, doxorubicin, cisplatin, and sorafenib.^[58]^

It was demonstrated that ubenimex efficiently increased the cytotoxicity of tested anticancer drugs by further inducing ROS generation, apoptosis, and cell cycle arrest in human hepatocellular carcinoma (HCC) cell lines HuH7, and PLC/PRF/5.^[58]^ Subsequently, deeper *in vitro* and *in vivo* investigations into the potential combination of ubenimex and 5‐FU were performed, showing improved efficiency and reduced toxicity than 5‐FU administered alone.^[59]^ These data, along with other studies on the synergistic anticancer activity of 5‐FU with the aminopeptidase N inhibitor (*S*)‐4‐methyl‐2‐(3‐(naphthalen‐1‐yl‐methyl)ureido)‐pentanoic acid hydroxyamide,^[60]^ led to novel 5‐FU/ubenimex codrugs.^[61]^ In the first 5‐FU/ubenimex mutual prodrug, named BC‐01 (Table 3), and synthesized by Jiang and co‐workers in 2016,^[61a]^ parent compounds were linked through a hydroxymethyl group easily cleaved after administration, as supported by preliminary stability studies in human plasma. Biological *in vitro* and *in vivo* studies demonstrated that conjugating 5‐FU with ubenimex could be an efficient strategy for optimizing the pharmacological profile of 5‐FU. Indeed, BC‐01 showed high anticancer, anti‐metastasis and anti‐angiogenic effects, while the cytotoxicity towards healthy cells was decreased compared with 5‐FU alone. Based on this work, further chemical modifications were performed to ameliorate the codrug's plasma stability and the CD13 inhibitory activity. To this purpose, the same research group more recently designed and synthesized new 5‐FU/ubenimex codrugs **7 a**–**e** by optimizing the structure of BC‐01.^[61b]^ In particular, the two active promoieties were linked through different connecting chains, as shown in Table 3.

**Table 3:** Antiproliferative activities and chemical structure of BC01 and 5‐FU/ubenimex codrugs **7 a**–**e**.

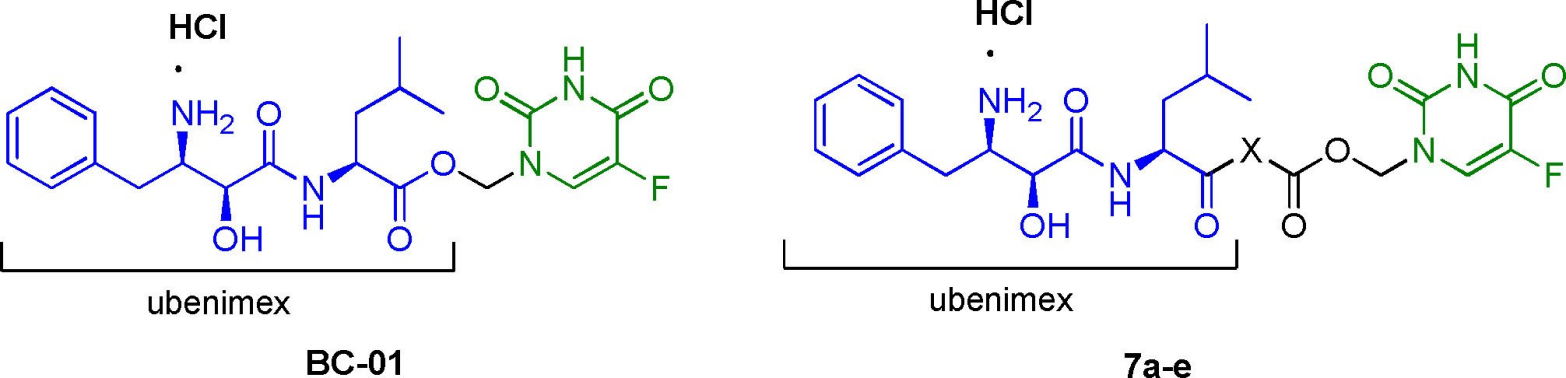			
Compd.	X	Cytotoxicity [IC_50_, μM]^[a]^	
		PLC/PRF/5	ES‐2
BC‐01	–	18.37	26.14
**7 a**	−NHCH_2_−	16.52	23.49
**7 b**	−NHCH(CH_3_)−	24.18	25.31
**7 c**	−NH(CH_2_)_2_−	58.96	130.56
**7 d**	−NH(CH_2_)_3_−	52.98	110.09
**7 e**	−NH(CH_2_)_5_−	60.08	104.48
5‐FU	–	35.66	71.47
Ubenimex	–	>500	>500
Ubenimex+5‐FU	–	22.81	37.05

[a] Data from Ref. [61b].

In this study, the mutual prodrugs approach successfully led to a more potent CD13 inhibition compared with both ubenimex and previously reported BC01, suggesting an advantage for selectively targeting cancer cells overexpressing CD13. Specifically, the introduction of an aliphatic central chain attached to the carbonyl group of ubenimex improved the plasma stability and prevented an early release of ubenimex and 5‐FU.

Furthermore, *in vitro* and *in vivo* studies revealed a good pharmacological profile for conjugates **7 a**–**e**, especially for **7 a**, which was the most potent compound among the series, and showed significant *in vivo* anti‐metastasis and lifespan extension effects compared to 5‐FU, ubenimex and 5‐FU/ubenimex coadministration.^[61b]^

### 2.4 5‐FU/oxaliplatin

**Figure 5:**
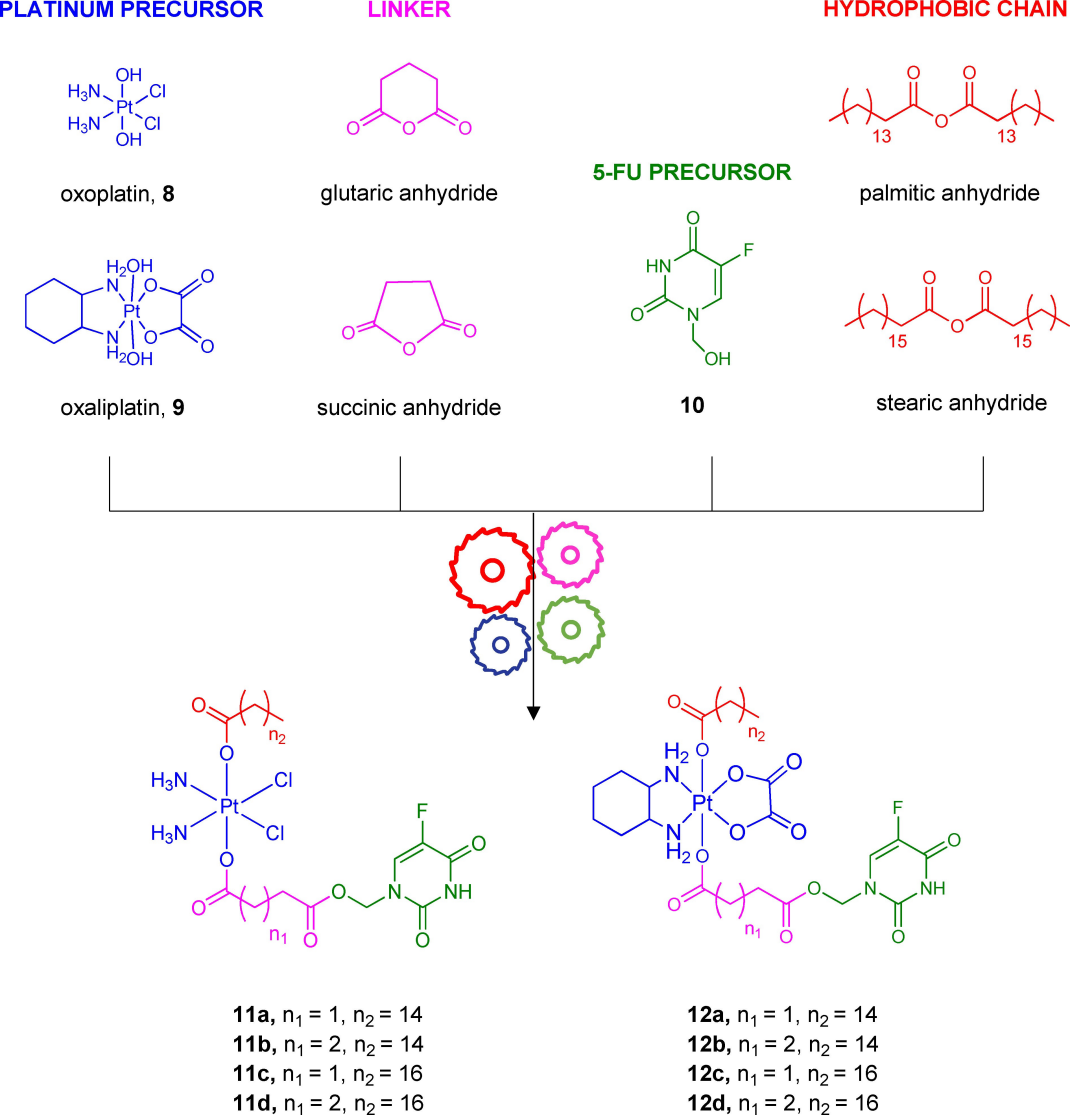
Chemical structure of codrugs **11 a**–**d**, **12 a**–**d**, their precursors **8**–**10**, their linkers, and hydrophobic chains.

Combining platinum drugs with fluoropyrimidines, such as 5‐FU, represents an ongoing attempt against colon cancer, which is the third most common type of cancer worldwide.^[62]^ Platinum‐based drugs have been widely used as alkylating antineoplastic agents over the last forty years.^[63]^ The first FDA‐approved platinum drug was cisplatin in the 1970s, followed by new generations of platinum antitumor agents (carboplatin, oxaliplatin, nedaplatin, and lobaplatin).^[64]^ Upon cellular uptake, platinum‐based drugs interact with their cellular target, the DNA, and coordinate to guanine bases, generating platinum−DNA adducts. The following distortion and DNA damage inhibit DNA replication. In addition, platinum‐based drugs bind to RNA, interfering with transcriptional processes, and inducing apoptosis.^[65]^ Oxaliplatin is a bifunctional alkylating agent belonging to the third generation of platinum‐based drugs.^[66]^ It is used as a first‐line treatment for patients with metastatic colorectal cancer in combination with 5‐FU. However, oxaliplatin‘s high risk of severe systemic toxicity and intrinsic or acquired resistance are still its main disadvantages. Multiple cellular mechanisms appear to be responsible for the loss of response to oxaliplatin. Among the most important ones, there are aberrations in transport, epigenetic profile, drug detoxification mechanisms, DNA injury response, and repair pathways.^[67]^ Furthermore, clinical trials evaluated the efficacy of adjuvant treatments that combine oxaliplatin with other chemotherapeutic drugs, such as 5‐FU, irinotecan, folinic acid, and leucovorin against different types of tumors, especially metastatic cancer.^[68]^ Although adjuvant chemotherapy‘s primary purpose is to increase anticancer efficacy, managing systemic side effects and avoiding multidrug‐resistances (MDR) are necessary for improving response rates and survival outcomes. In this setting, some platinum (IV) prodrugs that release platinum (II) agents (cisplatin, carboplatin, oxaliplatin) have been recently developed.^[69]^ Platinum (IV) compounds showed desirable properties in comparison with platinum (II) drugs because the two additional axial ligands in platinum (IV) complexes, which are released on reduction in cancer tissues, and may provide several pharmacokinetic advantages, such as kinetic stability, lipophilicity, tumor‐targeting or synergistic effect.^[70]^ In an effort to overcome the pharmacokinetic limits of oxaliplatin in combination with 5‐FU, a series of platinum (IV)/5‐FU codrugs have been synthesized and evaluated *in vitro* and *in vivo*.^[71]^ The chemical structures of the potential low‐toxic mutual prodrugs **11 a**–**d, 12 a**–**d**, named fuplatin, are shown in Figure 5.

The synthesis of fuplatin compounds **11 a**–**d, 12 a**–**d** was performed by esterification reactions. Platinum (IV) **8**, **9**, and 5‐FU precursors (**10**) were coupled using succinic anhydride or glutaric anhydride to obtain succinyl or glutaryl linkers, respectively. Additionally, the introduction of long hydrophobic chains (i. e., palmitoyl or stearyl chain) was performed to increase the codrugs′ lipophilicity and improve their cellular uptake. As expected, compounds **11 a**–**d, 12 a**–**d** showed higher cytotoxic activity than the oxaliplatin and 5‐FU administered alone or in combination under adjuvant regiment. These results were obtained from seven cancer cell lines (HeLa, MCF‐7, CaCo‐2, LoVo, HCT‐116, A549, MRC‐5). Furthermore, their potential synergistic effect was investigated since both 5‐FU and platinum metabolites should target and damage the DNA. The most potent compound **12 a** exhibited a lower IC_50_ value (0.13 μM) than those of mono‐therapy (IC_50_ 5‐FU=7.56 μM, and IC_50_ oxaliplatin=8.34 μM) or co‐therapy (IC_50_ oxaliplatin/5‐FU=5.40 μM) in HCT‐116 cells. Also, high selectivity against cancer cells, massive intracellular uptake, and improved survival rates (100 % for **12 a** *vs*. 57.1 % for oxaliplatin and 50 % for oxaliplatin/5‐FU co‐therapy) were observed.^[71]^ The stability of **12 a** was studied through HPLC analysis using a metabolic extract of HCT‐116 cells, confirming the ability of **12 a** to release 5‐FU and oxaliplatin, and the latter then converted into the active Pt(II) congener because of the reduction condition of cells. Interestingly, the intracellular dual‐activity of **12 a** and a synergistic effect between 5‐FU and oxaliplatin were assessed thanks to flow cytometry studies. As a result, the cell cycle inhibition caused by **12 a** seemed to occur through 5‐FU and oxaliplatin effects, which blocked both the S and G2 phases.

Besides, *in vivo* studies on male NOD/SCID mice suggested improved tumor growth inhibition and less toxic effects for **12 a** than 5‐FU, oxaliplatin, and 5‐FU/oxaliplatin coadministration. These results show that it is worth focusing on existing compounds (e. g., platinum‐based agents) to develop new potential 5‐FU codrugs with a more tolerable toxicological profile.

### 2.5 5‐FU/parthenolide

Parthenolide (PTL) is a germacrene sesquiterpene lactone, first found in *Tanacetum parthenium*, known as feverfew.^[72]^ Since ancient times, the plant was used in traditional herbal medicine against fever, migraine, arthritis, and gastrointestinal diseases.^[73]^ It took its name from the anti‐inflammatory and antitumor properties attributed to its main bioactive component, PTL. This secondary metabolite has been widely studied for its multi‐pharmacological potential. The most interesting effects are the anti‐inflammatory, redox‐modulating, epigenetic, antioxidant, and antiproliferative activities due to the modulation of multiple targets.^[74]^ Moreover, PTL is currently the object of clinical studies,^[75]^ and it is of considerable interest for its ability to selectively kill cancer cells without affecting normal ones. PTL's anticancer effect was studied at the molecular level on breast cancer cells, where it induces ROS generation, M phase cell cycle arrest, and apoptosis.^[76]^ Furthermore, PTL was cytotoxic against malignant stem cells, which play a central role in tumor pathogenesis and relapse.^[77]^ Synergistic action between 5‐FU and PTL was also investigated in CRC cells (SW620 cells), proving that adjuvant treatment with 5‐FU and PTL can overcome 5‐FU resistance and inhibits cell growth more efficiently through the enhancement of apoptosis.^[78]^ Based on these *in vitro* and *in vivo* evidences, new 5‐FU/PTL codrugs were developed with the main purpose of overcoming 5‐FU resistance.^[79]^ Table 4 displays the most representative and potent 5‐FU/PTL hybrids synthesized so far.

**Table 4:** Antiproliferative activities and chemical structures of PTL and PTL/5‐FU hybrids **13 a**–**e**.

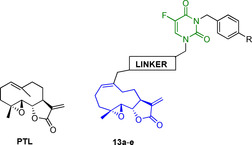				
Compd.	R	Linker	Cytotoxicity [IC_50_, μM]^[a]^	
			Bel‐7402	Bel‐7402/5‐FU
**13 a**	CF_3_	−OCO−	2.25	2.25
**13 b**	Cl	−OCO−	2.56	2.42
**13 c**	F	−OCO−	2.73	2.90
**13 d**	F	1,2,3‐triazole	4.91	4.86
**13 e**	H	−OCO(CH_2_)_2_CONH(CH_2_)_6_NHCO−	18.29	16.16
PTL	–	–	8.62	12.98
5‐FU	–	–	7.36	>400
PTL+5‐FU (1 : 1)	–	–	–	8.36

[a] Data from Ref. [79].

The majority of 5‐FU/PTL hybrids belonging to this series (16 out of 23) demonstrated increased cytotoxicity comparing their parent compounds 5‐FU and PTL against human hepatocellular carcinoma cell line (Bel‐7402) and 5‐FU resistant human hepatocellular carcinoma cell line (Bel‐7402/5‐FU). SAR studies were also performed to explore the effect of a wide variety of substituents on the benzyl group at *N* ^*3*^ **‐**5**‐**FU moiety. The most active conjugate was **13 a**, which bears a trifluoromethyl benzyl group. It showed 5.8‐fold improved activity than PTL, thus it was submitted for preliminary studies on its mechanism of action. To this extend, flow cytometry studies were performed and revealed that the proapoptotic activity of conjugate **13 a** was due to its ability to increase the levels of proapoptotic mediators, such as Bax, Bim, cytochrome C, caspase‐3, and caspase‐9. Furthermore, it was investigated the expression of proteins involved in the generation of drug resistance as efflux transporters, MDR1, ABCC1, and ABCG2, to detect how **13 a** can be useful to overcome 5‐FU resistance. As a result, **13 a** showed to significantly down‐regulate the expression of the previously mentioned proteins after 24 h treatment. These results suggested that 5‐FU codrugs might be useful not only for improving physicochemical and pharmacokinetic properties but also for increasing the drug uptake and overcoming 5‐FU drug resistance.

### 2.6 5‐FU/pentacyclic triterpenes

**Figure 6:**
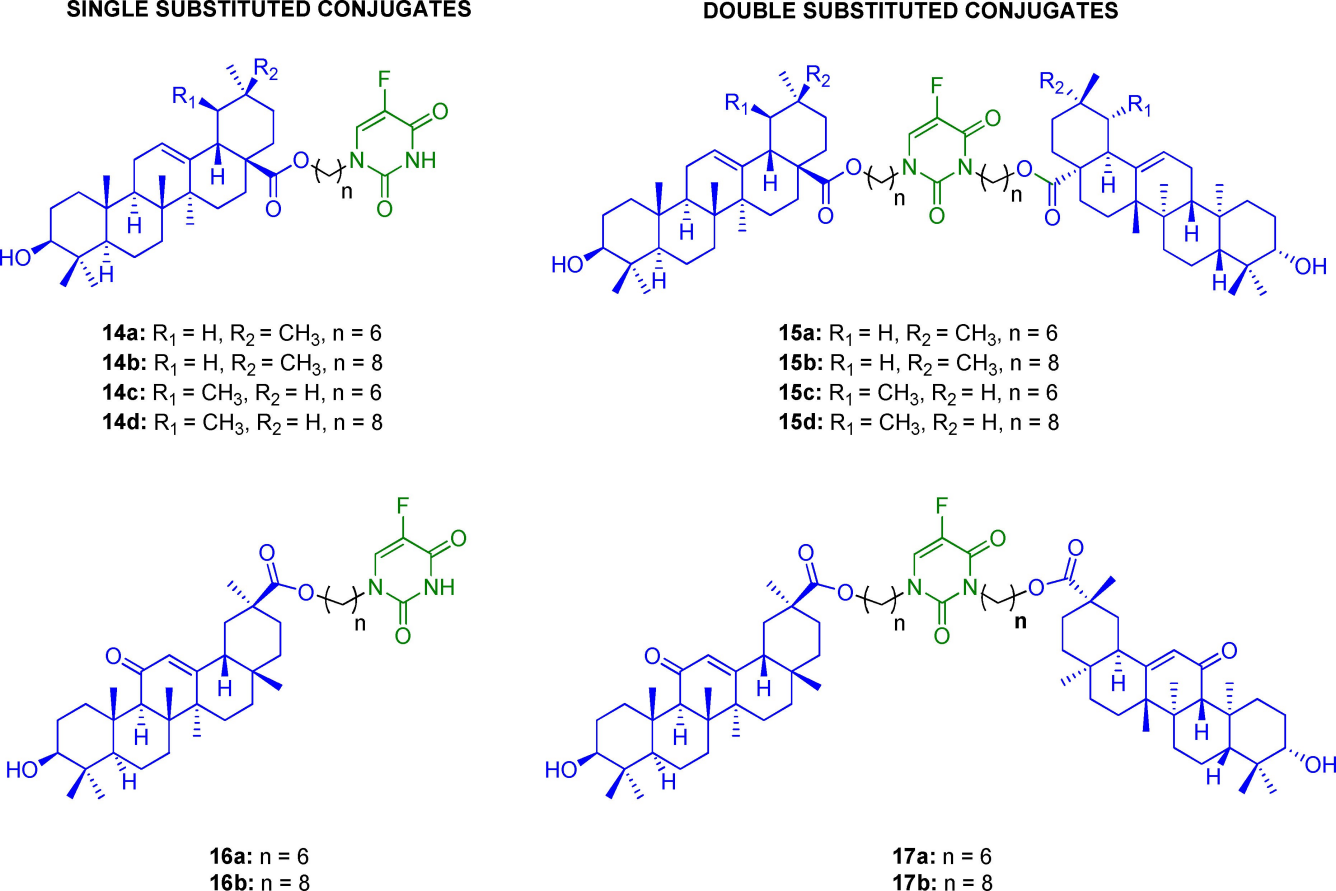
Chemical structure of conjugates **14 a**–**d**, **15 a**–**d**, **16 a**,**b**, and **17 a**,**b**.

Oleanolic, ursolic, and glycyrrhetinic acids (OA, UA, GA, respectively) are pentacyclic triterpenes present in many medicinal plants.^[80]^ These natural isoprenoids gained much attention as ecological tools to fight several pathological conditions.^[81]^ Indeed, many studies showed multifaceted effects of this group of compounds, such as antioxidant, anti‐inflammatory, antihepatodamage, antiviral, and anticancer effects.^[82]^ However, the mechanism of action responsible for their anticancer activity has not been fully clarified yet, because of the involvement of diverse signal pathways.^[83]^ Pharmacophore hybridization has been successfully used as a strategy to improve the antiproliferative and cytotoxic effects of pentacyclic triterpenes. For example, oleanolic acid dimers exhibited higher cytotoxicity and selectivity towards tumor cell lines than a single unit of pentacyclic triterpene and 5‐FU.^[84]^ Following this study, 5‐FU was conjugated with OA/UA/GA through single or double substitution to reduce 5‐FU side effects (Figure 6).^[85]^ The best results were obtained with single substituted conjugates **14 a**–**d**, **16 a**,**b**; in particular, compound **16 a** showed good antiproliferative activity, especially against MDR cell lines A549/T (IC_50_=20.73 μM) and Bel‐7402/FU (IC_50_=19.77 μM).

The most potent compound **16 a** was submitted to further studies to investigate its mechanism of action on A549 cell lines. Detection of intracellular calcium ions was performed after the administration of **16 a**, revealing that the conjugate was able to stimulate the intracellular influx of calcium, which acts as an essential cell signaling regulator.^[86]^ Also, **16 a** showed to induce ROS production, inhibiting the cell cycle at the G1 phase and inducing apoptosis through the activation of caspase‐3.^[85]^ Despite the good biological activity exhibited by **16 a**, the exact mechanism of 5‐FU release and its subsequent activation to active antimetabolite, which is required to exert the antiproliferative effect, is unclear due to the lack of *in vitro* or *in vivo* stability studies. Thus, for the sake of clarity, we can consider the 5‐FU/pentacyclic triterpenes as conjugates rather than mutual prodrugs.

### 2.7 5‐FU/HO‐1 inhibitor

Heme oxygenase (HO) is a family of cytoprotective enzymes that degrades heme yielding three metabolites: carbon monoxide (CO), ferrous iron (Fe^2+^), and biliverdin (BV), then converted to bilirubin (BR).^[87]^

**Figure 7:**
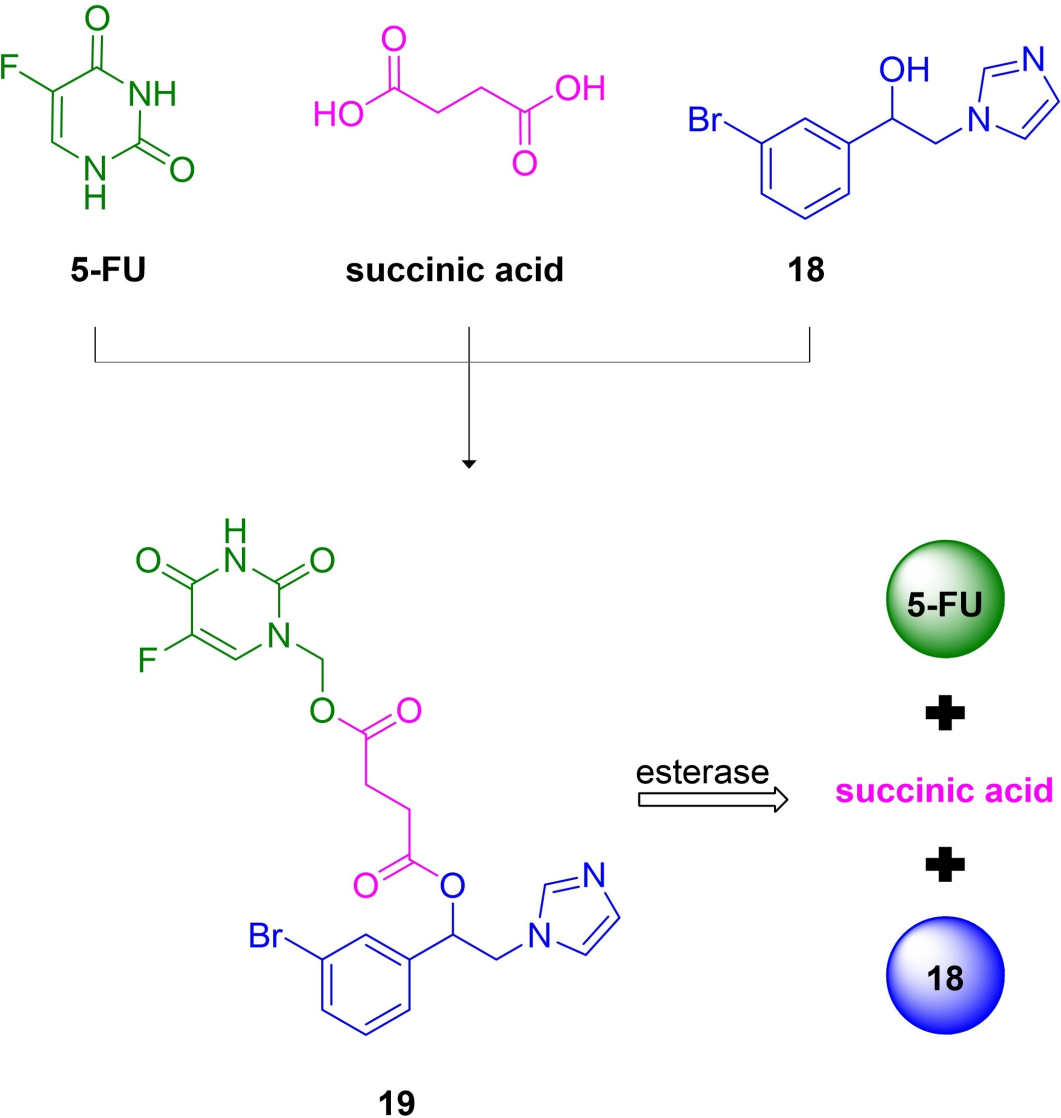
Chemical structure of 5‐FU/HO‐1 mutual prodrug **19**, its precursors 5‐FU and HO‐1 inhibitor **18**, and the proposed biotransformation process.

Three different isoforms of HO have been identified. The inducible isoform is the first one, named HO‐1, predominantly expressed in the spleen and the liver under physiological conditions. An increased amount of HO‐1 is reported in response to stressful factors, including oxidative phenomena, heavy metals, hypoxia, heme itself, and UV radiations.^[88]^ Indeed, HO‐1 provides a cytoprotective response thanks to both the degradation of free heme, which can be toxic at high concentrations, and to the beneficial properties of its catabolites. Although HO‐1 induction plays an essential role under several pathological conditions,^[89]^ growing pieces of evidence have highlighted HO‐1 overexpression in tumor development and progression.^[90]^ For this reason, a large set of HO‐1 inhibitors have been designed and developed,^[91]^ showing efficient antiproliferative effects on several tumor models, especially in combination with other anticancer drugs.^[92]^ For example, HO‐1 imidazole‐based aryloxy alkyl inhibitors combined with imatinib, a tyrosine kinase inhibitor, showed antitumor activity and potential beneficial effects in overcoming imatinib resistance in chronic myeloid leukemia, leading to the development of a series of HO‐1/imatinib multitarget ligands.^[93]^ Taking advantage of this evidence, very recently, 5‐FU was conjugated with a potent azole‐based HO‐1 inhibitor (**18**) (HO‐1 IC_50_=0.4 μM and HO‐2 IC_50_=32.0 μM),^[94]^ using succinic acid as a spacer (Figure 7).^[95]^

The resulting hybrid **19** showed decreased HO‐1 inhibitory activity with respect to the parent inhibitor **18**, however, this expected result was consistent with previous SARs performed on azole‐based compounds.

The *in silico* prediction of physicochemical, ADME, and toxicity properties of **19** indicate suitable drug‐like properties. Moreover, *in vitro* chemical stability studies suggested that hybrid **19** was stable at gastric pH values, thus potentially suitable for oral administration. Furthermore, appropriate enzymatic cleavage occurred in porcine esterase solution, confirming that the succinyl linker was a valid biodegradable spacer, able to release the two active components, **18** and 5‐FU. In addition, preliminary cytotoxicity studies on prostate (DU‐145) and lung (A549) cancer cells were performed. Interestingly, hybrid **19** showed remarkable antiproliferative activity in a dose‐dependent manner, similar to that obtained with the administration of 5‐FU alone and the simultaneous administration of 5‐FU and **18**. These preliminary data, together with the lower toxicity of **19** on a healthy human lung epithelial cell line (BEAS‐2B) compared to 5‐FU, provided evidence to support the development of new 5‐FU/HO‐1 mutual prodrugs that may be able to overcome some of 5‐FU limits.

Table 5 summarizes the main features and studies performed on mutual prodrugs described in Section 2.

**Table 5:** Summary of mutual prodrugs of 5‐FU with improved biological activity.

Compd.	Type of hybrid	Linker	Study	Outcomes	Ref.
**1 a,b**	5‐FU/HDAC	carbamate	molecular docking, *in vitro* antiproliferative assay, *in vitro* HDAC inhibition, *in vitro* stability assay	↓ side effects	[42]
**4 a**–**f, 5 6 a**–**f**	5‐FU/deoxy‐ podophyllotoxin	amino acid, diamine	*in vitro* cytotoxicity assay, immunofluorescence, *in vitro* HUVECs tube formation assay	↑ antiproliferative activity	[50]
**7 a**–**e**	5‐FU/ubenimex	carbamate	*in vitro* antiproliferative assay, *in vitro* HUVECs tube formation assay, *in vivo* antitumor activity assay	Antiproliferative activity	[61b]
**11 a**–**d, 12 a**–**d**	5‐FU/oxaliplatin	succinyl, glutaryl	*in vitro* antiproliferative assay, measurement of water‐octanol partition coefficient, apoptosis analysis, stability in PBS/DMF buffer, *in vivo* antitumor activity	↑ antiproliferative activity ↓ side effects	[71]
**13 a**–**e**	5‐FU/ parthenolide	ester, triazole	*in vitro* cytotoxicity assay, cell apoptosis assay, western blot assay, nuclear morphology assay, drug accumulation assay	overcoming 5‐FU resistance	[79]
**14 a**–**d, 15 a**–**d, 16 a,b, 17 a,b**	5‐FU/ pentacyclic triterpenes	alkyl	*in vitro* cytotoxicity assay, intracellular Ca^2+^ generation, detection of intracellular ROS, Cell cycle assay, detection of caspase‐3/8	↑ antiproliferative activity	[85]
**19**	5‐FU/HO‐1 inhibitor	succinyl	*in vitro* cytotoxicity assay, measurement of HO‐1 and HO‐2 enzymatic activities*, in vitro* stability in acid, neutral, basic buffer solution and in porcine esterase solution, *in silico* prediction of physicochemical, ADME, and toxicity properties	comparable antiproliferative activity ↓ side effects	[95]

## 3 Mutual Prodrugs of 5‐FU for Targeted Drug Delivery

Significant efforts have focused on developing potential tools to target cancer cells selectively.^[96]^ One of the main challenges for developing an efficient anticancer therapy is reducing the dosage and side effects. With this in mind, many 5‐FU‐based mutual prodrugs have been developed by conjugating 5‐FU with a carrier for targeting specific cancer tissues or cellular organelles. This strategy involves the coupling of 5‐FU moiety with a carrier, most frequently a peptide.^[97]^ The resulting codrug can then be activated after the binding with receptors at the site of action. The following section discusses a few examples of 5‐FU‐based mutual codrugs designed for drug delivery purposes.

### 3.1 5‐FU/aspartic acid oligopeptides

**Figure 8:**
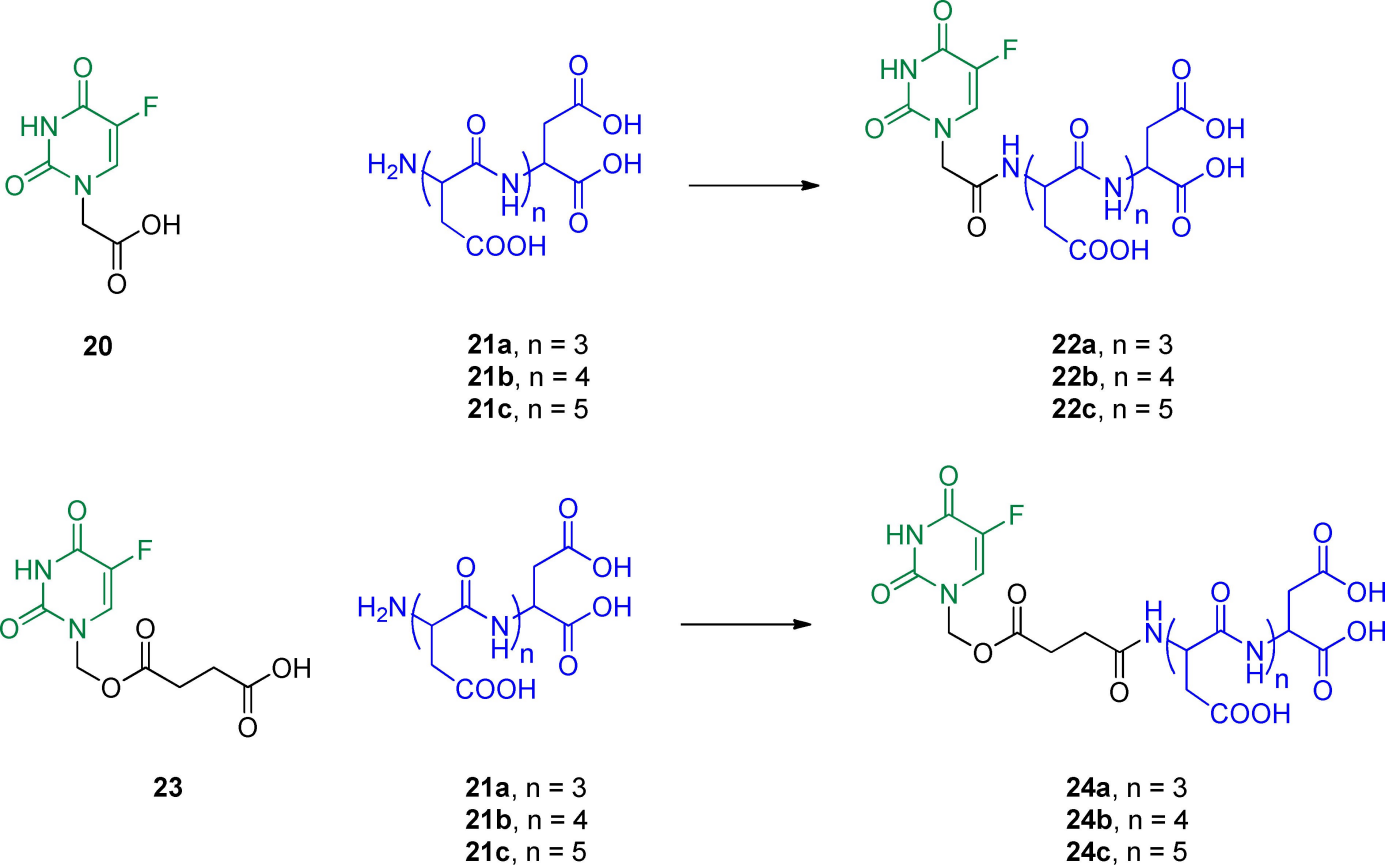
Chemical structure of codrugs **22 a**–**c** and **24 a**–**c** and their precursors **20**, **21 a**–**c** and **23**.

Acidic oligopeptides containing aspartic acid (Asp) sequences have been recognized as a promising class of molecules for targeting the bone with a selective mode of action.^[98]^ Indeed, these acidic oligopeptides′ main advantage is their ability to target hydroxyapatite (HAP), which is one of the main mineral constituents of vertebrate bones.^[99]^ This approach inspired the development of prodrugs, activated after targeting HAP for selective drug delivery to bone.^[100]^ Following the strategy mentioned above, Asp oligopeptides were linked to 5‐FU to obtain mutual prodrugs **22 a**–**c** and **24 a**–**c** (Figure 8) that effectively target the bone and reduce the high dose and frequent administration generally required for the treatment of bone tumor.^[101]^ Conjugates **22 a**–**c** were synthesized through the direct coupling of the acetic acid functionalized 5‐FU (**20**) with the bone targeting oligopeptides **21 a**–**c**. On the other hand, conjugates **24 a**–**c** were obtained by introducing a succinate chain (easy to cleave) at the *N* ^*1*^ of 5‐FU (compound **23**) to link Asp oligopeptides to 5‐FU.

To evaluate the effectiveness of the new mutual prodrugs, *in vitro* and *in vivo* studies were performed.^[101a]^ The affinity of the conjugates with HAP was measured to estimate the bone targeting ability of the compounds. Results from *in vitro* HAP binding assay showed that the bone binding property was conferred by the presence of the oligopeptides **21 a**–**c**, since 5‐FU alone, did not bind the bone. The best results were achieved with compounds **22 c** and **24 c** due to the presence of six amino acid residues interacting with the mineral components of bone tissues. Besides, *in vitro* drug release profile was studied to prove that codrugs were hydrolyzed to release the two different moieties. This study demonstrated an efficient release of 5‐FU from the oligopeptide portion under physiological conditions, especially for conjugates **24 a**–**c**, confirming that the succinate linkage was suitable for the enzymatic cleavage of conjugates. Moreover, MTT assay showed that the conjugates were cytotoxic in two cancer cell lines, human epithelial carcinoma (HeLa) and human osteosarcoma cell line (MG63). These outcomes, supported by suitable pharmacokinetic parameters highlighted by *in vivo* pharmacokinetic and biodistribution studies, proved that coupling 5‐FU with Asp oligopeptides could be a promising approach for achieving a high accumulation of different chemotherapeutic agents to the bone, limiting the dosage and severe side effects.

### 3.2 5‐FU/mitochondria‐targeting lipophilic cations

**Figure 9:**
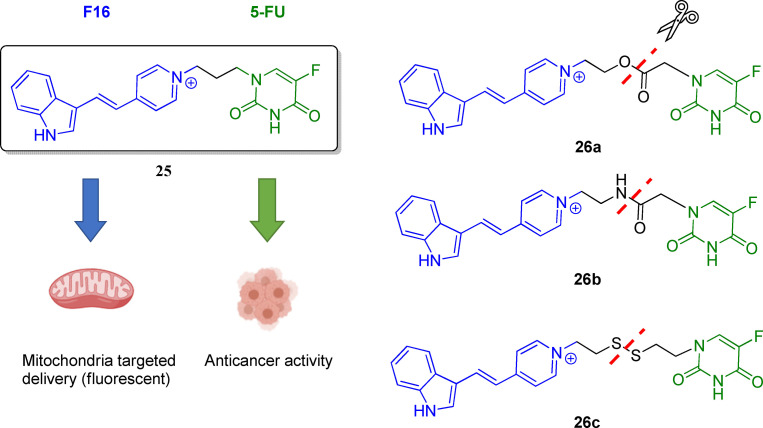
Chemical structure of the first developed 5‐FU/F16 mutual codrug **25** and its derivatives **26 a**–**c**.

Mitochondrial‐targeting anticancer agents represent promising chemical tools to enhance cytotoxicity towards malignant cells and as mitochondria‐targeted nanocarrier systems for anticancer agent delivery.^[102]^ Indeed, mitochondria are involved in various pathways regulating cells′ survival, stress, and death.^[103]^ Mitochondria are pluripotent organelles with a prominent role in energy production and are essential for anabolic and catabolic processes.^[104]^ Interestingly, a relationship between mitochondria and tumorigenesis has also been recognized, inspiring the scientific community to regard these organelles as a target (e. g., mitochondrial antiapoptotic proteins) for future anticancer treatments.^[105]^ For instance, several hallmarks of cancer are related to mitochondria alterations.^[106]^ In order to develop mutual prodrugs that selectively target mitochondria, 5‐FU was conjugated with (*E*)‐4‐(1*H*‐indol‐3‐ylvinyl)‐*N*‐methylpyridinium iodide (F16) (Figure 9).^[107]^ Specifically, F16 is a delocalized lipophilic cationic (DLC) small compound with mitochondria‐targeted and fluorescent properties. Interestingly, F16 might be attached to another pharmacophore, such as 5‐FU, to achieve selective cytotoxicity in cancer cells by triggering apoptosis, as proved in a wide variety of cancer cells.^[108]^ In addition, in the context of developing antitumor probes, F16 might provide desirable optical properties being fluorescent in the visible region.^[108]^

The first synthesized 5‐FU/F16 hybrid was compound **25** (Figure 9).^[109]^ Unexpectedly, conjugate **25** did not show increased cytotoxicity against the human gastric carcinoma SGC‐7901 cell line, with respect to healthy cells. Therefore, to optimize the structure of compound **25**, three different kinds of hydrolyzable bridges (i. e., ester, amide, and disulfide) were used to link the two moieties, leading to 5‐FU/F16 mutual prodrugs **26 a**–**c** (Figure 9).^[107]^ The stability of these conjugates was studied, showing that compounds **25**, **26 a**–**c** were stable in PBS for 72 h. Furthermore, thanks to the fluorescence properties conferred to the conjugates by F16, cellular uptake, and localization studies were performed.

Results proved that linking 5‐FU with F16 increased the cytotoxicity against cancer cells; in particular, conjugate **26 a** showed high mitochondrial uptake and caused tumor cell death, reducing the cytotoxicity against healthy cells GES‐1. This study showed that attaching 5‐FU to probes, such as DLCs, can be an advantageous method to reduce 5‐FU side effects by delivering the chemotherapeutic agent to the mitochondria of tumor cells.

### 3.3 5‐FU/integrin‐targeting peptides

**Figure 10:**
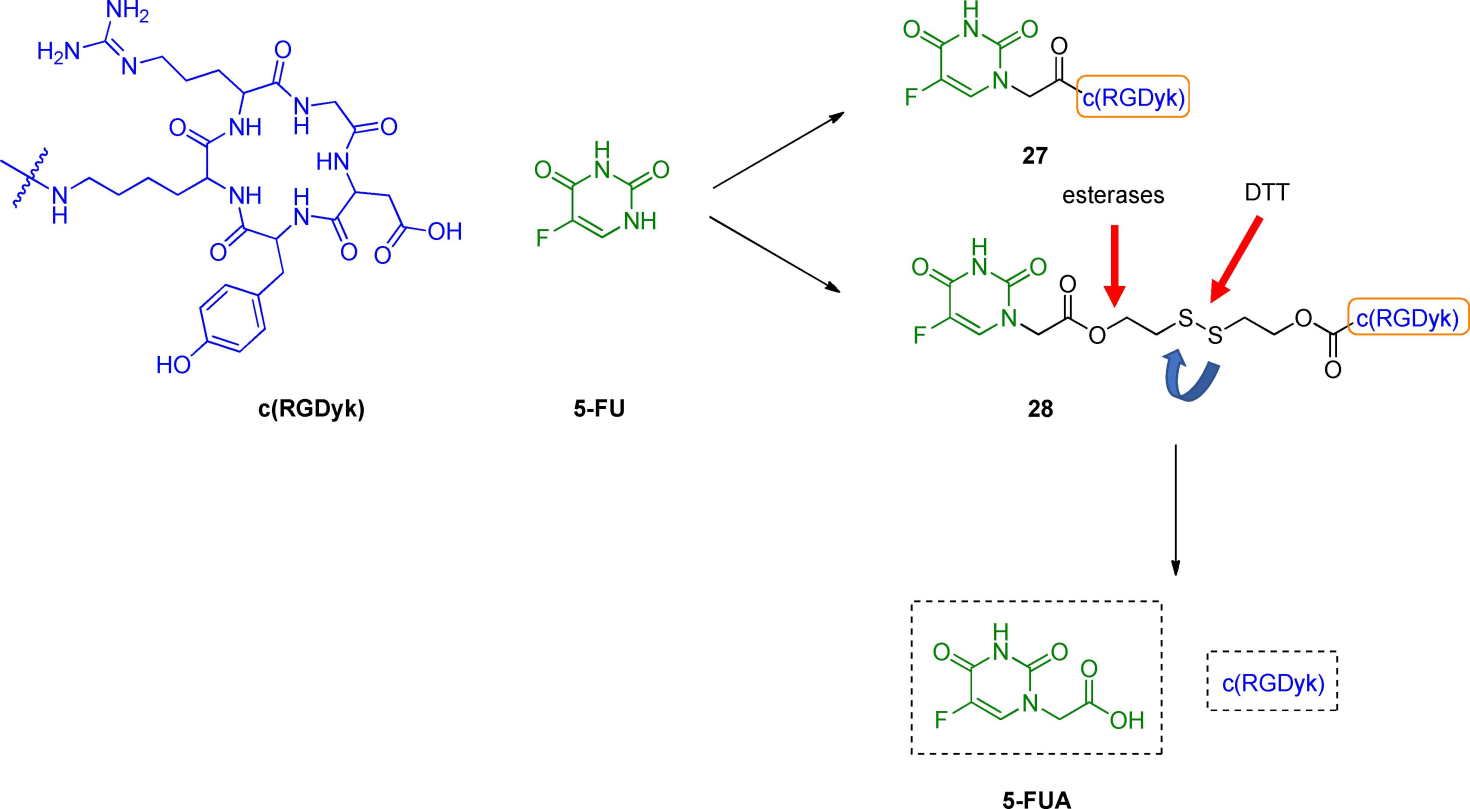
Development of c(RGDyK)‐based 5‐FU codrugs **27** and **28**.

The usage of the arginine‐glycine‐aspartic acid (RGD) sequence has been regarded as a promising strategy for improving drug selectivity.^[110]^ Importantly, this sequence has been recognized as the cell attachment site for several proteins. Different integrins recognize the tripeptide RGD within their ligands;^[111]^ for instance, α_v_β_3_ and α_v_β_5_ integrin receptors are over‐expressed in tumor tissues, playing a crucial role in the initiation, progression, and metastasis of tumors.^[112]^ On this basis, RGD peptides have been designed and developed for providing both an antagonist effect against integrin receptors and intracellular selective delivery of therapeutics through an integrin receptor‐mediated endocytosis.^[113]^ Following these pieces of evidence, c(RGDyK)‐based conjugates have been reported for integrin targeted chemotherapy.^[114]^ 5‐FU‐based mutual prodrugs were developed by using c(RGDyK) peptide as drug delivery carrier for 5‐FU inside cancer cells after the binding with integrin receptors.^[115]^ Particularly, 5‐FU and c(RGDyK) peptide were linked through an amide bond or disulfide linker to obtain new codrugs **27** and **28** (Figure 10).

The antiproliferative activity of **27** and **28** was investigated towards five different cell lines, (i. e., prostate adenocarcinoma PC3, ovarian SKOV3, lung adenocarcinoma A549, breast adenocarcinoma MCF7 and MDA‐MB‐321 cells), with various integrin α_v_β_3_ expression using the MTT assay. Improved growth inhibitory effect was observed for conjugate **28** compared with the parent 5‐FU in all the selected cell lines, except for PC3 cells. Different results were obtained for compound **27**, which showed to be effective only against MDA‐MB‐321 cells.

^1^H‐NMR, MS, and HPLC analysis were performed to understand the possible mechanism that leads to the release of 5‐fluorouracil acetic acid (5‐FUA), a widely studied 5‐FU prodrug.^[116]^ The latter might be released after the cleavage at two different levels: at the ester functionality by plasma esterases or by reducing the disulfide bond by biologically available thiols. To investigate this second mechanism of release, dithiothreitol (DTT) was used, showing an early release of 5‐FUA from **28**. As a consequence, the active metabolite 5‐FUA might be released before the endocytic process. These findings suggested that the anticancer activity of conjugates **27** and **28** was highly dependent on their stability and cell integrin expression levels. More in‐depth linker investigation and measurement of the integrin expression level could give further information about the metabolite's activity for selected cancer cell lines. Next, *in vivo* characterization should be considered to evaluate their pharmacological efficacy and potential clinical applications.

Table 6 lists the main features of mutual prodrugs described in Section 3.

**Table 6:** Summary of mutual prodrugs of 5‐FU for targeted drug delivery.

Compd.	Type of hybrid	Linker	Study	Outcomes	Ref.
**22** **c**, **24 a**–**c**	5‐FU/aspartic acid oligopeptides‐based	succinate	HAP binding study, drug release study, *in vitro* cytotoxicity assay, *in vivo* biodistribution in mice tissue	targeting the bone antiproliferative activity	[101a]
**26 a**–**c**	5‐FU/F16	carboxylic, amidic, disulfide	*in vitro* cytotoxicity assay, detection of cellular uptake, colocalization assay, detection of apoptosis and cell cycle, *in vitro* measurement of intracellular oxidation level, stability study in PBS or DMEM medium	targeting mitochondria ↑ antiproliferative activity	[107]
**27**, **28**	5‐FU/c(RGDyK)	amide, disulfide	*in vitro* cytotoxicity assay, chemostability study, 5‐FUA release assay	integrin targeted chemotherapy ↑ antiproliferative activity	[115]

## 4 Conclusions

The prodrug design has been successfully used to improve the pharmacokinetic profile of many existing drugs. Among the different types of prodrugs, mutual prodrugs are of particular interest to medicinal chemists. This approach has shown to be potentially advantageous for chemotherapy regimens, which are often highly invasive causing systemic collateral effects. Notably, the strategy has been largely applied to 5‐FU, a well‐known antimetabolite of great interest in treating many tumors.

Following evidence showing beneficial effects of 5‐FU coadministration with other agents, efforts have been devoted to conjugate 5‐FU, through a cleavable spacer, to active promoieties.

This review highlights representative examples of 5‐FU‐based mutual prodrugs, where the coupled promoiety gave one of the following advantages: *i)* achieve a synergistic/additive effect, ii) exert site‐specific drug delivery.

Interestingly, in many cases, preliminary *in vitro* and *in vivo* studies showed increased biological effects for 5‐FU‐based mutual prodrugs compared to the antiproliferative activity of a single active moiety. On the other hand, coupling 5‐FU with a drug‐delivery moiety led to successful attempts with high drug uptake in cancer tissues or mitochondria, suggesting that this could be also a useful strategy for overcoming the lack of site‐specificity. Importantly, to develop effective mutual prodrugs, preliminary stability and release studies are mandatory to validate the strategy and investigate the molecule‘s behavior after administration.

Although promising advances in increased antiproliferative activity and targeted drug delivery have already been achieved, some limitations related to chemical stability, administration route, and bioavailability of mutual prodrugs still need to be faced.

## Conflict of interest

The authors declare no conflict of interest.

## Biographical Information

*Valeria Ciaffaglione is currently a Ph.D. student in Chemical Sciences at the Department of Drug and Health Sciences, University of Catania, working on a project in medicinal chemistry under the supervision of Prof. Loredana Salerno. She graduated in Pharmacy with honors in July 2018 at the same Institution. Her research activity is focused on the synthesis and characterization of novel compounds that modulate heme oxygenase‐1 endowed with potential anticancer activity, including hybrids and mutual prodrugs*.

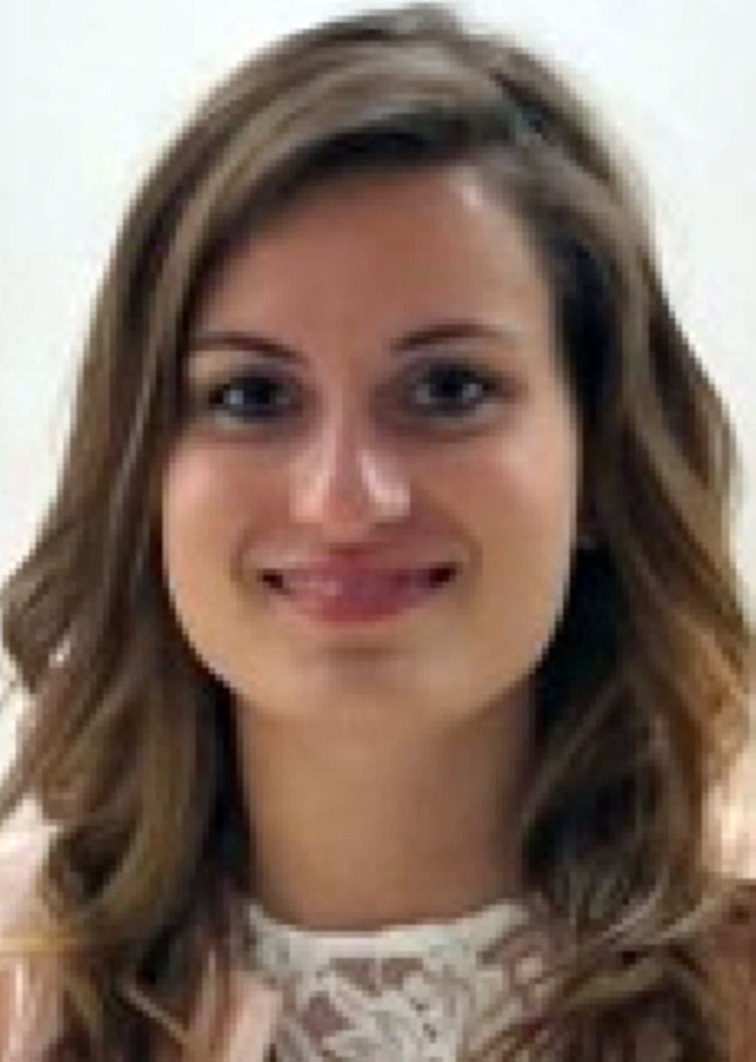

## Biographical Information

*Maria N. Modica is an Associate Professor in Medicinal Chemistry at the Department of Drug and Health Sciences, University of Catania. She graduated in Pharmacy at the University of Catania, in 1989, discussing a thesis in medicinal chemistry. In 1994, she received a Ph.D. in Pharmaceutical Sciences from the same Institution. Her research interests include the synthesis of ligands for the 5‐HT_1A_ and 5‐HT_7_ serotonin, α_1_‐adrenergic, and endothelin receptors*.

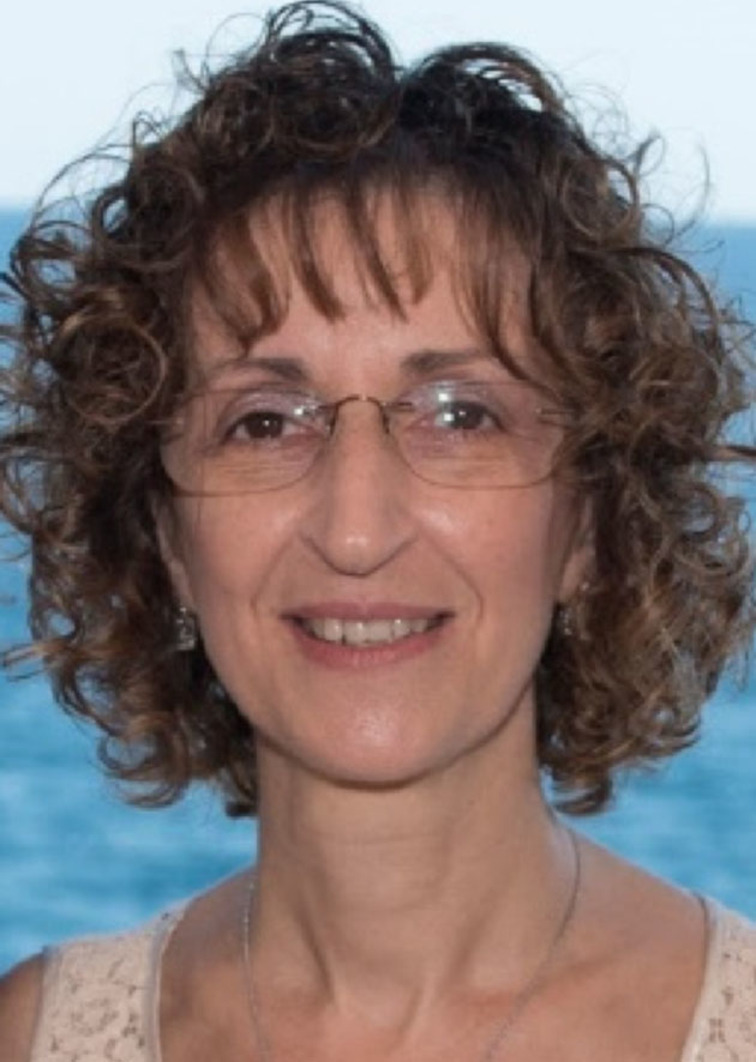

## Biographical Information

*Valeria Pittalà is currently an Associate Professor in Medicinal Chemistry at the Department of Drug and Health Sciences, University of Catania. She attained an M.Sc. in Chemistry and Pharmaceutical Technologies and completed a Ph.D. in Pharmaceutical Sciences at the University of Catania (Italy). Her current research focuses on the design, synthesis, and structure−activity relationship studies of small molecules acting towards different biological targets including enzymes (i. e., heme oxygenase) and GPCRs (i. e., ET and 5‐HT receptors)*.

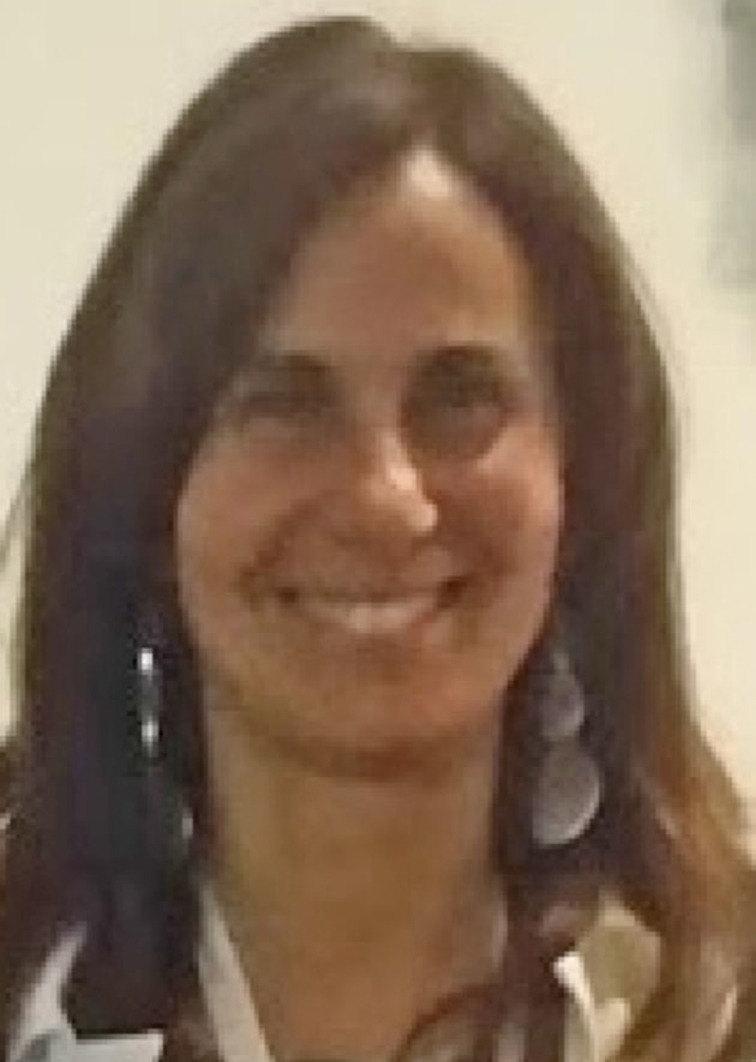

## Biographical Information

*Giuseppe Romeo is currently an Associate Professor in Medicinal Chemistry at the Department of Drug and Health Sciences, University of Catania. In 1985, he received his degree in Pharmacy with honors from the University of Catania. During his career, he has been mainly involved in research project dealing with the design, synthesis, and characterization of novel heterocyclic compounds endowed with potential pharmacological activity. The main topics of his research include the development of selective ligands for the α1‐adrenoceptor subtypes and for the serotonin 5‐HT_1A_ and 5‐HT_7_ receptors*.

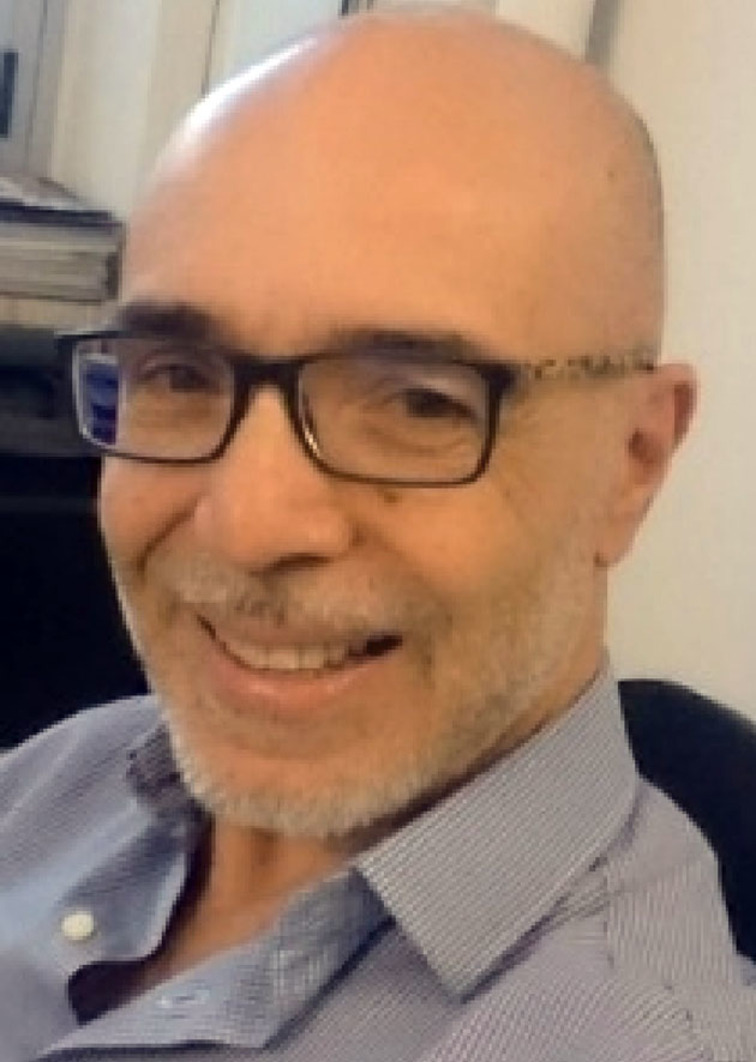

## Biographical Information

*Loredana Salerno is an Associate Professor in Medicinal Chemistry at the Department of Drug and Health Sciences, University of Catania. She obtained the degree in Pharmacy in 1986 from the University of Catania, and in 1993, the Ph.D. in Pharmaceutical Sciences. Her research activity includes the development of ligands for serotonergic and adrenergic receptor ligands, enzymatic inhibitors of nitric oxide synthase and heme oxygenase‐1 as antitumor drugs and modified natural compounds as inducers of heme oxygenase‐1 useful in stress‐induced diseases*.

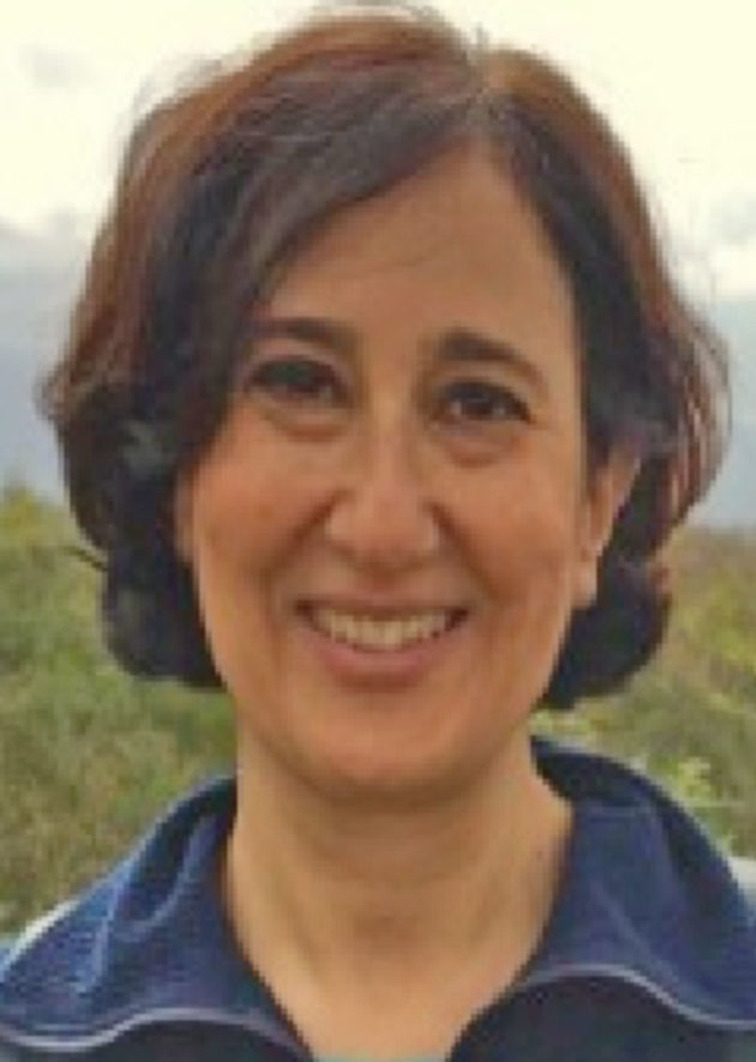

## Biographical Information

*Sebastiano Intagliata is a Research Associate in the Department of Drug and Health Sciences, University of Catania. He earned his M.S. in Chemistry and Pharmaceutical Technology, and his Ph.D. in Pharmaceutical Sciences from the University of Catania. He was a Post‐Doctoral Associate at the University of Mississippi, and then at the University of Florida. His current research focuses on the design and synthesis of novel cytotoxic agents such as heme oxygenase 1 inhibitors, and σRs ligands and the development of their hybrids or codrugs with anticancer agents*.